# A novel technique to study the solutions of time fractional nonlinear smoking epidemic model

**DOI:** 10.1038/s41598-024-54492-0

**Published:** 2024-02-20

**Authors:** K. Pavani, K. Raghavendar

**Affiliations:** Department of Mathematics, School of Advanced Sciences, Vellore Institute of Technology, Vellore, 632014 India

**Keywords:** Caputo–Fabrizio derivative, Natural transform decomposition method, Caputo derivative, Atangana–Baleanu–Caputo derivative, Smoking model, Mathematics and computing, Applied mathematics

## Abstract

The primary goal of the current work is to use a novel technique known as the natural transform decomposition method to approximate an analytical solution for the fractional smoking epidemic model. In the proposed method, fractional derivatives are considered in the Caputo, Caputo–Fabrizio, and Atangana–Baleanu–Caputo senses. An epidemic model is proposed to explain the dynamics of drug use among adults. Smoking is a serious issue everywhere in the world. Notwithstanding the overwhelming evidence against smoking, it is nonetheless a harmful habit that is widespread and accepted in society. The considered nonlinear mathematical model has been successfully used to explain how smoking has changed among people and its effects on public health in a community. The two states of being endemic and disease-free, which are when the disease dies out or persists in a population, have been compared using sensitivity analysis. The proposed technique has been used to solve the model, which consists of five compartmental agents representing various smokers identified, such as potential smokers *V*, occasional smokers *G*, smokers *T*, temporarily quitters *O*, and permanently quitters *W*. The results of the suggested method are contrasted with those of existing numerical methods. Finally, some numerical findings that illustrate the tables and figures are shown. The outcomes show that the proposed method is efficient and effective.

## Introduction

There are many applications for the broad field of mathematical biology. Researchers in this discipline are concentrating on the representation of various disease types with controls in the form of mathematical models. Brownlee^1^ spearheaded the development of mathematical biology in 1909, with a focus on the theory of fortuitous events. Moreover, he outlined the fundamental laws of epidemic propagation^2^ in 1912. The finer points of the epidemic investigation were explored by Kermark and McKendrik^3^ in 1927. Later, many scholars discussed various models for numerous other diseases^4–9^. The development of social habits, including alcohol consumption^10^, obesity epidemics^11^, cocaine use^12^, smoking habit^13^, and numerous others, have all been studied using this type of model. One of these models that has drawn the most interest from researchers in recent years is the smoking model. By way of the lungs, the smoke is breathed. The smoking epidemic is the world’s most hazardous pandemic. 50% of users died as a result of smoking. About 60 million individuals die from smoking diseases each year. The number of deaths has significantly increased during the past few decades.

Smoking is the world’s most hazardous habit. Smoking has an impact on a variety of bodily organs, and as a result, it contributes to more than a million fatalities worldwide. Smoking is a key contributor to lung and heart attacks globally. A smoker has a 70% higher risk of having a heart attack than a nonsmoker. Similar to this, smokers have an incidence rate of lung cancer that is 10% higher than nonsmokers. There are 200 million female smokers and 900 million male smokers worldwide. There is a smoking-related death every 6 s. Short-term smoking’s main side effects include foul breath, stained teeth, increased blood pressure, and coughing. In recent years, mouth cancer, throat cancer, lung cancer, heart disease, and stomach ulcers have been the main side effects of long-term smoking.

The frequency of puffs and other materials affects the chemical composition of smoke. Nicotine is the primary cause of wrinkles because it disrupts the neurological system, causes an increase in heartbeat and blood pressure, and causes the little blood vessels that are the foundation of wrinkles to constrict. Due to carbon monoxide, the amount of oxygen in the lungs is reduced. Hydrogen cyanide destroys the little hairs that act as the natural cleanser of the lungs. Smoke also contains cadmium, lead, nickel, and arsenic. Smoke contains some pesticides, such as dichloro-diphenyl-trichloroethane. A harmful chemical found in smoke is the main cause of skin and lung cancer. A smoker consumes 1–2 mg of nicotine per cigarette each day. Thus, 5 cigarettes would require at least 5–10 mg of nicotine. Smoking has negative effects that are not only felt by the smoker but also by others around them. Smoking has a negative impact on human fertility^14^. It contributes to 22% of deaths per year.

An individual who smokes will live 12–13 years less than an individual who does not. Smoking kills numerous individuals around the world every day, according to reports from the World Health Organization. Many scientists, mathematicians, and medical professionals are working to restrict smoking in order to protect human life. Due to these reasons, the smoking model has been studied by numerous scholars using various techniques; we list here a few recent works such as the Laplace-Adomian decomposition method (LADM)^15^, homotopy perturbation technique (HPM)^16^, reduced differential transform technique^17^, Atangana–Toufik method^18^, and fractional differential transformation technique^19^.

Fractional calculus, which focuses on the operation of fractional order differentiation and integration, is a generalisation of classical calculus. In the nineteenth century, mathematicians working with fractional calculus invented fractional differential equations, fractional geometry, and fractional dynamics. Almost all branches of science employ fractional calculus. It is used to model both engineering and physical processes. Standard mathematical models of integer order frequently fail to operate as intended. Because of this, fractional calculus significantly advanced the sciences of chemistry, biology, image processing, and mechanics. Numerous physical issues can be resolved using fractional calculus. The system demonstrates numerous issues, including history and nonlocal effects, by utilising integer-order derivatives. Riemann–Liouville (R–L) fractional derivatives and Caputo fractional order were the main foundations of all the studies. These derivatives have a singular kernel.

This singularity would not allow us to explain the full memory of physical structures. Because of this restriction, novel definitions were presented in the studies^20–24^. As nonsingular kernels that meet their requirements, these new definitions have a significant influence. The fundamental distinction between the derivatives named Caputo (C), Caputo–Fabrizio (CF), and Atangana–Baleanu–Caputo (ABC) is the method used to calculate each of them: C uses a power law, CF uses an exponential decay law, and ABC uses the Mittag–Leffler law. Many scholars have focused their efforts in recent years on studying fractional nonlinear partial differential equation (PDE) solutions using a variety of methodologies, including the new iterative transform method^25^, homotopy perturbation transform technique^26^, reduced differential transform technique^27^, homotopy perturbation Sumudu transform technique^28^, q-homotopy analysis transform method (q-HATM)^29^, Laplace residual power series technique^30^, and the finite difference method^31^.

The goal of this study is to use natural transform decomposition method (NTDM) to solve the nonlinear time fractional smoking model. Rawashdeh and Maitama^32^ introduced the NTDM for a category of nonlinear PDEs. NTDM is devoloped by combining the Adomian decomposition method and natural transform (NT). The relationship between the natural transform and other integral transforms can be found in^33^. The NTDM efficiently reduces round-off errors without the need for perturbation, prescribed assumptions, discretization, or linearization. The proposed method is distinguished by its simplicity and effectively resolves specific limitations encountered in previous investigations. A fundamental limitation of the HPM method is the necessity to solve the functional equation in every iteration, which can present difficulties and consume a significant amount of time. The variational iteration method exhibits inherent accuracy in computing the Lagrange multiplier, corrective function, and stationary conditions for problems involving fractional orders. In contrast to the conventional Adomian method, the proposed approach eliminates the need to calculate fractional derivatives or fractional integrals in the recursive formula. This modification enhances the ability to calculate the values of the terms in the series. The homotopy analysis method requires the identification of the parameter *h* which is tricky. However, the NTDM is characterised by a straightforward concept, yet it demonstrates high efficiency in solving nonlinear fractional differential equations. To put it more specifically, it may be utilized to resolve a system of fractionally-ordered linear and nonlinear PDEs. The solutions provided by this technique might be precise or approximative and is based on a quick convergence series. This framework is effective for determining the analytical solution of partial differential equations of fractional order, but only under certain initial conditions in time. Based on our understanding, the suggested approach appears to be viable for resolving initial value problems; however, it may not be well-suited for problems that incorporate boundary constraints. As a result, this framework is exclusively intended for transient state problems in which the initial quantity distribution is provided. The efficacy and reliability of NTDM have prompted a recent surge in authors exploring and examining the resultant implications of a diverse array of differential equations, for example the Klein–Gordon equation^34^, Kawahara and modified Kawahara equations^35^, Zakharov–Kuznetsov equation^36,37^, coupled fractional Ramani equations^38^, Burgers–Huxley equation^39^, and Swift–Hohenberg equation^40^ have been resolved using the proposed approach. NT is gaining popularity by coupled with different techniques to solve the non linear equations, for example the authors in^41^ applied natural-homotopy perturbation method, which is a generalization of homotopy-Sumudu transformation technique. Recently, NT is employed to study fuzzy fractional wave equation^42^.

The overview of the article is presented as follows: A model description is provided in “Model description” section. The equilibrium point and stability are covered in “Equilibrium point and stability” section. The NT and other preliminary concerns involving the singular and non-singular kernels derivatives are covered in “Preliminaries” section. The current technique is presented in “The methodology of NTDM” section. The existence, uniqueness and convergence are included in “Existence and uniqueness” section. The fractional smoking model’s solution is described in “Illustrative example” section. Results and discussion are provided in “Numerical results and discussion” section. The concluding remarks are briefed in “Conclusion” section.

## Model description

By creating mathematical models and examining their dynamical behaviors, it is a crucial and effective technique to comprehend biological issues. In this study, the system consisting of five equations involving nonlinear differentials characterizing the smoking pandemic model is considered. Let $N(\tau )$ denote the overall population, at time $\tau$ . We break down the population $N(\tau )$ into five categories in order to better comprehend it: potential smokers $V (\tau )$, occasional smokers $G(\tau )$, smokers $T(\tau )$, temporarily quitting smokers $O(\tau )$, and permanently quitting smokers $W(\tau )$. The suggested smoking model^43^ is given as a system of nonlinear differential equations with the following coefficients:

1 $$\begin{aligned} \left\{ \begin{aligned} \frac{dV}{d\tau }&=\alpha -\epsilon \,V\,T-\vartheta \,V, \\ \frac{dG}{d\tau }&=\epsilon \,V\,T-\varepsilon _{1}\,G-\vartheta \,G, \\ \frac{dT}{d\tau }&=\varepsilon _{1}\,G+\varepsilon _{2}\,T\,O-(\vartheta +\rho )T,\\ \frac{dO}{d\tau }&=-\varepsilon _{2}\,T\,O-\vartheta \,O+\rho (1- \sigma )T,\\ \frac{dW}{d\tau }&=\sigma \,\rho \,T-\vartheta \,W. \end{aligned}\right. \end{aligned}$$

The recruitment rate for potential smokers is represented by $\alpha$, the effective contact rate between smokers and potential smokers is represented by $\epsilon$, the natural death rate is represented by $\vartheta$, the rate of quitting smoking is represented by $\rho$, the remaining percentage of smokers who permanently quit smoking is represented by $\sigma$, the rate at which occasional smokers become regular smokers are represented by $\varepsilon _{1}$, and the contact rate between smokers and temporary quitters who return to smoking is represented by $\varepsilon _{2}$. Table 1 provides information on the parameters employed in the system of Eq. (1).

The utilisation of fractional-order differential equations with time delay has been commonly employed in the field of biology in recent decades. Biological systems modelled using fractional-order differential equations produce more realistic and accurate outcomes in capturing the hereditary and memory characteristics of the system, as opposed to models based on integer-order differential equations. The majority of mathematical models of biological systems include a form of enduring historical memory^44^. Fractional order extension of the model (1) was initially investigated in^45,46^. Fractional differential equations are used to demonstrate the genuine biphasic decrease behaviour of disease infection, albeit at a slower rate. The fractional differential equation system is described by the following.

2 $$\begin{aligned} \begin{aligned} D^{\mu }_{\tau }V&=\alpha -\epsilon \,V\,T-\vartheta \,V, \\ D^{\mu }_{\tau }G&=\epsilon \,V\,T-\varepsilon _{1}\,G-\vartheta \,G, \\ D^{\mu }_{\tau }T&=\varepsilon _{1}\,G+\varepsilon _{2}\,T\,O-(\vartheta +\rho )T,\\ D^{\mu }_{\tau }O&=-\varepsilon _{2}\,T\,O-\vartheta \,O+\rho (1-\sigma )T,\\ D^{\mu }_{\tau }W&=\sigma \,\rho \,T-\vartheta \,W, \end{aligned} \end{aligned}$$

with the initial conditions

3 $$\begin{aligned} V(0)=K_{1}, ~ G(0)=K_{2}, ~ T(0)=K_{3}, ~ O(0)=K_{4}, ~ W(0)=K_{5}. \end{aligned}$$

The system is qualitatively examined in two different methods, namely endemic equilibrium and disease free equilibrium.

**Table 1:** The parameters utilized in system (2), along with a description of their particular values.

Parameters	Descriptions	Values (unit: 1/ time)
$\alpha$	The *V* is the rate of recruitment	1
$\epsilon$	Effective contact rate between *T* and *V*	0.14
$\vartheta$	Rate of natural death	0.05
$\rho$	Rate of smoking cessation	0.8
$\sigma$	Remaining smoking percentage who successfully gave up smoking	0.1
$\varepsilon _{1}$	Rate of transition from occasional smokers to habitual smokers	0.002
$\varepsilon _{2}$	Interaction ratio between smokers and transient abstainers who resume smoking	0.0025

## Equilibrium point and stability

Analysing the differential equations presented in Eq. (2) can provide valuable understanding of the propagation of smoking and the potential methods for restricting its spread. The reproductive number is a crucial tool for analysing a model of this nature. The basic reproduction number, represented as $R_0$, and is defined as the number of new infections produced by a typical infective individual in a susceptible population at a disease free equilibrium. In the scenario of disease-free equilibrium, a value of $R_0<1$ indicates that the disease will ultimately disappear. Conversely, in the case of endemic equilibrium, a value of $R_0>1$ indicates that the disease will propagate throughout the population.

In system (2) we consider equilibrium point^47^, we take

4 $$\begin{aligned} D^{\mu }_{\tau }V(\tau )=D^{\mu }_{\tau }G(\tau )=D^{\mu }_{\tau }T(\tau ) =D^{\mu }_{\tau }O(\tau )=D^{\mu }_{\tau }W(\tau )=0. \end{aligned}$$

We achieved equilibria that were disease-free.

$$\begin{aligned} E_{0}=(V,0,T,0,0), \end{aligned}$$

as well as the system being in endemic equilibrium

$$\begin{aligned} E^{*}=(V^{*},G^{*},T^{*},O^{*},W^{*}), \end{aligned}$$

where

5 $$\begin{aligned} V^{*}=\frac{\alpha }{\epsilon \,T+\vartheta }, ~ G^{*}=\frac{\epsilon \,\alpha \,T}{(\epsilon \,T+\vartheta )(\varepsilon _{1}+\vartheta )}, ~ O^{*}=\frac{\rho (1-\sigma )T}{\varepsilon _{2}\,T+\vartheta }, ~ W^{*}=\frac{\sigma \,\rho \,T}{\vartheta }. \end{aligned}$$

### Theorem 1

^15^ The diseases free equilibrium $E_{0}$ is locally asymptotically stable for $R_{0}<1$ otherwise unstable.

Consider the Jacobian matrix as $$J=\begin{bmatrix} -\epsilon T-\vartheta &{} 0 &{} -\epsilon V&{} 0&{}0\\ \epsilon T &{} -\varepsilon _{1}-\vartheta &{} \epsilon V &{} 0&{}0\\ 0&{} \varepsilon _{1} &{} \varepsilon _{2} O-(\vartheta +\rho )&{} 0 &{}0\\ 0 &{}0 &{} -\varepsilon _{2} O+\rho (1-\sigma )&{} -\varepsilon _{2} T-\vartheta &{} 0\\ 0&{}0&{}\rho \sigma &{} 0 &{} -\vartheta \\ \end{bmatrix}.$$ Since the Jacobian matrix is $J=F-Q$ then the matrix *F* and *Q* can be written as $$F=\begin{bmatrix} 0 &{} 0 &{} 0 &{} 0&{}0\\ 0 &{} 0 &{} 0 &{} 0&{}0\\ 0&{} \varepsilon _{1} &{} 0&{} 0 &{}0\\ 0 &{}0 &{}0&{} 0&{} 0\\ 0&{}0&{}0 &{} 0 &{} 0 \\ \end{bmatrix},$$

$$Q=\begin{bmatrix} \epsilon T+\vartheta &{} 0 &{} \epsilon V&{} 0&{}0\\ -\epsilon T &{} \varepsilon _{1}+\vartheta &{} -\epsilon V &{} 0&{}0\\ 0&{} -\varepsilon _{1} &{} -\varepsilon _{2} -O+(\vartheta +\rho )&{} 0 &{}0\\ 0 &{}0 &{} \varepsilon _{2} O-\rho (1-\sigma )&{} \varepsilon _{2} T+\vartheta &{} 0\\ 0&{}0&{}-\rho \sigma &{} 0 &{} \vartheta \\ \end{bmatrix}.$$

We know that $B=FQ^{-1}$ and using the relation $|B-\lambda I|=0$ for the eigen value $\lambda$, we get

$$\begin{aligned} \lambda =\frac{\varepsilon _{1}\epsilon \vartheta V}{(\varepsilon _{1}+\vartheta )(\vartheta +\rho )(\vartheta +\epsilon T)}, \end{aligned}$$

by substituting the values of each parameters, we get $0<\lambda <1$, which shows the reproductive number $R_{0}<1$, so the constructed system is in diseases free state. Reproductive number in this system (2) is

$$\begin{aligned} R_{0}=\frac{\varepsilon _{1}\epsilon \vartheta V}{(\varepsilon _{1}+\vartheta )(\vartheta +\rho )(\vartheta +\epsilon T)}. \end{aligned}$$

We see that all the eigen values are negative only for $R_{0}<1$. Thus the disease free state is locally asymptotically stable for $R_{0}<1$, otherwise unstable.

**Sensitivity analysis of** $R_{0}$: The sensitivity of $R_{0}$ to each of its parameters is

$$\begin{aligned} \frac{\partial R_{0}}{\partial \varepsilon _{1}}&=\frac{\epsilon \vartheta ^{2}V}{(\varepsilon _{1}+\vartheta )^{2}(\vartheta +\rho )(\vartheta +\epsilon T)}\ge 0.\\ \frac{\partial R_{0}}{\partial \epsilon }&=\frac{\varepsilon _{1}\vartheta ^{2}V}{(\varepsilon _{1}+\vartheta )(\vartheta +\rho )(\vartheta +\epsilon T)^{2}}\ge 0.\\ \frac{\partial R_{0}}{\partial \rho }&=\frac{\varepsilon _{1}\epsilon \vartheta ^{2}V}{(\varepsilon _{1}+\vartheta )(\vartheta +\rho )^{2}(\vartheta +\epsilon T)}\le 0.\\ \frac{\partial R_{0}}{\partial \vartheta }&=\frac{\epsilon \vartheta ^{2}V[(\varepsilon _{1}+\vartheta )^{2}(\vartheta +\rho )(\vartheta +\epsilon T)]-\vartheta }{[(\varepsilon _{1}+\vartheta )(\vartheta +\rho )(\vartheta +\epsilon T)]^{2}}\ge 0. \end{aligned}$$

It can be seen that $R_{0}$ is most sensitive to change in parameters here $R_{0}$ is increasing with $\varepsilon _{1},\epsilon ,\vartheta$ and decreasing with $\rho$. In other words it found that the sensitivity analysis shows that prevention is better than quit smoking.

## Preliminaries

In this section, we will present some fractional derivative definitions along with the NT.

### Definition 4.1

^48^ The C derivative of $g\in C_{-1}^{q},~ q\in \mathbb {N}$ of order $\mu$ is as follows

6 $$\begin{aligned} D^{\mu }_{\tau }g(\tau )= {\left\{ \begin{array}{ll} \frac{d^{q}g(\tau )}{d\tau ^{q}}, ~~ \mu =q,\\ \frac{1}{\Gamma (q-\mu )}\int _{0}^{\tau }(\tau -\xi )^{q-\mu -1}g^{q}(\xi )d\xi , ~q-1<\mu \leqslant q. \end{array}\right. } \end{aligned}$$

### Definition 4.2

^20^ The CF derivative of the function $g(\tau )$ with order $\mu$ is represented as

7 $$\begin{aligned} {}^{CF}_{}D_{\tau }^{\mu }g(\tau )=\frac{1}{1-\mu }\int _{0}^{\tau }g^{\prime }(\zeta )~ \textrm{exp}\Big (\frac{-\mu (\tau -\zeta )}{1-\mu }\Big ) d\zeta ,~\tau \ge 0. \end{aligned}$$

### Definition 4.3

^49^ The ABC derivative of the function $g(\tau )$ with order $\mu$ is defined

8 $$\begin{aligned} {}^{ABC}_{}D_{\tau }^{\mu }g(\tau )=\frac{B[\mu ]}{1-\mu }\int _{0}^{\tau }g^{\prime }(\zeta ) E_{\mu }\Big (\frac{-\mu (\tau -\zeta )^{\mu }}{1-\mu }\Big ) d\zeta , \end{aligned}$$

here $E_{\mu }$ denotes the Mittag–Leffler function and $B[\mu ]$ is a normalization function.

### Definition 4.4

^50^ On employing the NT of the function $g(\tau )$ is stated as

9 $$\begin{aligned} N^{+}[g(\tau )]=\int _{0}^{\infty } e^{-s\tau }g(\varphi \tau )d\tau , ~~\varphi ,s> 0. \end{aligned}$$

### Definition 4.5

^51^ On employing the NT of C derivative is defined as

10 $$\begin{aligned} N^{+}\left[ {}^{C}_{0}D_{\tau }^{\mu }\varphi (\tau )\right] =\Big (\frac{s}{v}\Big )^{\mu } \left( N^{+}[\varphi (\tau )]-\frac{1}{s}\varphi (0)\right) . \end{aligned}$$

### Definition 4.6

^52^ On employing the NT of CF derivative is given as

11 $$\begin{aligned} N^{+}\left[ {}^{CF}_{0}D_{\tau }^{\mu }\varphi (\tau )\right] = \frac{1}{f(\mu ,s,v)}\left( N^{+}[\varphi (\tau )]-\frac{1}{s}\varphi (0)\right) , \end{aligned}$$

where $$f(\mu ,s,v)=1-\mu +\mu (\frac{v}{s})$$

### Definition 4.7

^51^ On employing the NT of ABC derivative is defined as

12 $$\begin{aligned} N^{+}\left[ {}^{ABC}_{0}D_{\tau }^{\mu }\varphi (\tau )\right] = \frac{1}{h(\mu ,s,v)}\left( N^{+}[\varphi (\tau )]-\frac{1}{s}\varphi (0)\right) , \end{aligned}$$

where $$h(\mu ,s,v)=\frac{1-\mu +\mu \Big (\frac{v}{s}\Big )^{\mu }}{B[\mu ]}$$.

## The methodology of NTDM

We study the given system of fractional nonlinear PDEs with the initial conditions utilizing singular and nonsingular kernel derivatives as given below to explain the fundamental concept of this approach.

**NTDM**$_{C}$: In view of Eq. (10) and initial conditions (3), we obtain

13 $$\begin{aligned} \begin{aligned} \Big (\frac{s}{v}\Big )^{\mu }\left[ N^{+}(V(\tau ))-\frac{40}{s}\right] =&N^{+}[\alpha -\epsilon \,V\,T-\vartheta \,V],\\ \Big (\frac{s}{v}\Big )^{\mu }\left[ N^{+}(G(\tau ))-\frac{10}{s}\right] =&N^{+}[\epsilon \,V\,T-\varepsilon _{1}\,G-\vartheta \,G],\\ \Big (\frac{s}{v}\Big )^{\mu }\left[ N^{+}(T(\tau ))-\frac{20}{s}\right] =&N^{+}[\varepsilon _{1}\,G+\varepsilon _{2}\,T\,O-(\vartheta +\rho )T],\\ \Big (\frac{s}{v}\Big )^{\mu }\left[ N^{+}(O(\tau ))-\frac{10}{s}\right] =&N^{+}[-\varepsilon _{2}\,T\,O-\vartheta \,O+\rho (1-\sigma )T],\\ \Big (\frac{s}{v}\Big )^{\mu }\left[ N^{+}(W(\tau ))-\frac{5}{s}\right] =&N^{+}[\sigma \,\rho \,T-\vartheta \,W]. \end{aligned} \end{aligned}$$

Operating with the inverse NT on (13), we have

14 $$\begin{aligned} \begin{aligned} V(\tau )=&N^{-1}\left[ \frac{40}{s}+\Big (\frac{v}{s}\Big )^{\mu }N^{+}\left[ \alpha -\epsilon \,V\,T-\vartheta \,V\right] \right] ,\\ G(\tau )=&N^{-1}\left[ \frac{10}{s}+\Big (\frac{v}{s}\Big )^{\mu }N^{+}\left[ \epsilon \,V\,T-\varepsilon _{1}\,G-\vartheta \,G\right] \right] ,\\ T(\tau )=&N^{-1}\left[ \frac{20}{s}+\Big (\frac{v}{s}\Big )^{\mu }N^{+}\left[ \varepsilon _{1}\,G+\varepsilon _{2}\,T\,O-(\vartheta +\rho )T\right] \right] ,\\ O(\tau )=&N^{-1}\left[ \frac{10}{s}+\Big (\frac{v}{s}\Big )^{\mu }N^{+}\left[ -\varepsilon _{2}\,T\,O-\vartheta \,O+\rho (1-\sigma )T\right] \right] ,\\ W(\tau )=&N^{-1}\left[ \frac{5}{s}+\Big (\frac{v}{s}\Big )^{\mu }N^{+}\left[ \sigma \,\rho \,T-\vartheta \,W\right] \right] . \end{aligned} \end{aligned}$$

The decomposition of the Adomian polynomials of nonlinear terms is as follow

15 $$\begin{aligned} V\,T=\sum _{k=0}^{\infty }A_{k}, ~~T\,O=\sum _{k=0}^{\infty }B_{k}. \end{aligned}$$

In the above Eq. (15), the $A_{k}$ and $B_{k}$ are both Adomian polynomials, and by using the formula^53^, they are computed.

In the below Eq. (16), $V( \tau )$, $G( \tau )$, $T( \tau )$, $O( \tau )$ and $W( \tau )$ are the unknown functions, which have the infinite series given by

16 $$\begin{aligned} V( \tau ) = \sum _{k=0}^{\infty } V_{k},~~ G( \tau ) = \sum _{k=0}^{\infty } G_{k},~~ T( \tau ) = \sum _{k=0}^{\infty } T_{k},~~ O( \tau ) = \sum _{k=0}^{\infty } O_{k},~~ W( \tau ) = \sum _{k=0}^{\infty } W_{k}. \end{aligned}$$

Making replacements of Eqs. (15) and (16) into (14), we have

17 $$\begin{aligned} \begin{aligned} \sum _{k=0}^{\infty } V_{k}=&N^{-1}\left[ \frac{40}{s}\right] +N^{-1}\left[ \Big (\frac{v}{s}\Big )^{\mu }N^{+}\left[ \alpha -\epsilon \,\sum _{k=0}^{\infty }\sum _{j=0}^{k}V_{j}(T_{k-j})-\vartheta \,\sum _{k=0}^{\infty }V_{k}\right] \right] ,\\ \sum _{k=0}^{\infty } G_{k}=&N^{-1}\left[ \frac{10}{s}\right] +N^{-1}\left[ \Big (\frac{v}{s}\Big )^{\mu }N^{+}\left[ \epsilon \,\sum _{k=0}^{\infty }\sum _{j=0}^{k}V_{j}(T_{k-j})-\varepsilon _{1}\,\sum _{k=0}^{\infty }G_{k}-\vartheta \,\sum _{k=0}^{\infty }G_{k}\right] \right] ,\\ \sum _{k=0}^{\infty } T_{k}=&N^{-1}\left[ \frac{20}{s}\right] +N^{-1}\left[ \Big (\frac{v}{s}\Big )^{\mu }N^{+}\left[ \varepsilon _{1}\,\sum _{k=0}^{\infty }G_{k}+\varepsilon _{2}\,\sum _{k=0}^{\infty }\sum _{j=0}^{k}T_{j}(O_{k-j})-(\vartheta +\rho )\,\sum _{k=0}^{\infty }T_{k}\right] \right] ,\\ \sum _{k=0}^{\infty }O_{k}=&N^{-1}\left[ \frac{10}{s}\right] +N^{-1}\left[ \Big (\frac{v}{s}\Big )^{\mu }N^{+}\left[ \varepsilon _{2}\,\sum _{k=0}^{\infty }\sum _{j=0}^{k}T_{j}(O_{k-j})-\vartheta \,\sum _{k=0}^{\infty }O_{k}+\rho (1-\sigma )\,\sum _{k=0}^{\infty }T_{k}\right] \right] ,\\ \sum _{k=0}^{\infty }W_{k}=&N^{-1}\left[ \frac{5}{s}\right] +N^{-1}\left[ \Big (\frac{v}{s}\Big )^{\mu }N^{+}\left[ \sigma \,\rho \,\sum _{k=0}^{\infty }T_{k}-\vartheta \,\sum _{k=0}^{\infty }W_{k}\right] \right] . \end{aligned} \end{aligned}$$

From (17), we have

$$\begin{aligned} {}^{C}V_{0}=40,~~ {}^{C}G_{0}=10,~~ {}^{C}T_{0}=20,~~ {}^{C}O_{0}=10,~~ {}^{C}W_{0}=5,~~ \end{aligned}$$

$$\begin{aligned} {}^{C}V_{1}&= N^{-1}\left[ \Big (\frac{v}{s}\Big )^{\mu }N^{+}\left[ \alpha -\epsilon \,V_{0}\,T_{0}-\vartheta \,V_{0}\right] \right] ,\\ {}^{C}G_{1}&= N^{-1}\left[ \Big (\frac{v}{s}\Big )^{\mu }N^{+}\left[ \epsilon \,V_{0}\,T_{0}-\varepsilon _{1}\,G_{0}-\vartheta \, G_{0}\right] \right] ,\\ {}^{C}T_{1}&= N^{-1}\left[ \Big (\frac{v}{s}\Big )^{\mu }N^{+}\left[ \varepsilon _{1}\,G_{0}+\varepsilon _{2}\,T_{0}\,O_{0}-(\vartheta +\rho )\,T_{0}\right] \right] ,\\ {}^{C}O_{1}&= N^{-1}\left[ \Big (\frac{v}{s}\Big )^{\mu }N^{+}\left[ -\varepsilon _{2}\,T_{0}\,O_{0}-\vartheta \,O_{0}+\rho (1-\sigma )\,T_{0}\right] \right] ,\\ {}^{C}W_{1}&= N^{-1}\left[ \Big (\frac{v}{s}\Big )^{\mu }N^{+}\left[ \sigma \,\rho \,T_{0}-\vartheta \,W_{0}\right] \right] ,\\ {}^{C}V_{2}&= N^{-1}\left[ \Big (\frac{v}{s}\Big )^{\mu }N^{+}\left[ \alpha -\epsilon \,(V_{0}\,T_{1}+V_{1}\,T_{0})-\vartheta \,V_{1}\right] \right] ,\\ {}^{C}G_{2}&= N^{-1}\left[ \Big (\frac{v}{s}\Big )^{\mu }N^{+}\left[ \epsilon \,(V_{0}\,T_{1}+V_{1}\,T_{0})-\varepsilon _{1}\,G_{1}-\vartheta \, G_{1}\right] \right] ,\\ {}^{C}T_{2}&= N^{-1}\left[ \Big (\frac{v}{s}\Big )^{\mu }N^{+}\left[ \varepsilon _{1}\,G_{1}+\varepsilon _{2}\,(T_{0}\,O_{1}+T_{1}\,O_{0})-(\vartheta +\rho )\,T_{1}\right] \right] ,\\ {}^{C}O_{2}&= N^{-1}\left[ \Big (\frac{v}{s}\Big )^{\mu }N^{+}\left[ -\varepsilon _{2}\,(T_{0}\,O_{1}+T_{1}\,O_{0})-\vartheta \,O_{1}+\rho (1-\sigma )\,T_{1}\right] \right] ,\\ {}^{C}W_{2}&= N^{-1}\left[ \Big (\frac{v}{s}\Big )^{\mu }N^{+}\left[ \sigma \,\rho \,T_{1}-\vartheta \,W_{1}\right] \right] ,\\&\vdots \end{aligned}$$

18 $$\begin{aligned} \begin{aligned} {}^{C}V_{k+1}&= N^{-1}\Big [\Big (\frac{v}{s}\Big )^{\mu }N^{+}\Big [\alpha -\epsilon \,\sum _{j=0}^{k}V_{j}T_{k-j}-\vartheta \,V_{k}\Big ],~ k\ge 0, \\ {}^{C}G_{k+1}&= N^{-1}\Big [\Big (\frac{v}{s}\Big )^{\mu }N^{+}\Big [\epsilon \,\sum _{j=0}^{k}V_{j}T_{k-j}-\varepsilon _{1}\,G_{k}-\vartheta \,G_{k}\Big ],~ k\ge 0, \\ {}^{C}T_{k+1}&= N^{-1}\Big [\Big (\frac{v}{s}\Big )^{\mu }N^{+}\Big [\varepsilon _{1}\,G_{k}-\varepsilon _{2}\,\sum _{j=0}^{k}T_{j}O_{k-j}-(\vartheta +\rho )\,T_{k}\Big ],~ k\ge 0, \\ {}^{C}O_{k+1}&= N^{-1}\Big [\Big (\frac{v}{s}\Big )^{\mu }N^{+}\Big [-\varepsilon _{2}\,\sum _{j=0}^{k}T_{j}O_{k-j}-\vartheta \,O_{k}+\rho (1-\sigma )\,T_{k}\Big ],~ k\ge 0, \\ {}^{C}W_{k+1}&= N^{-1}\Big [\Big (\frac{v}{s}\Big )^{\mu }N^{+}\Big [\sigma \,\rho \,T_{k}-\vartheta \,W_{k}\Big ],~ k\ge 0. \end{aligned} \end{aligned}$$

Making replacements of Eq. (18) into (16), finally we obtain the series solution as

19 $$\begin{aligned} \begin{aligned} {}^{C}V(\tau )=&{}^{C}V_{0}+{}^{C}V_{1}+{}^{C}V_{2}+\cdots ~,\\ {}^{C}G(\tau )=&{}^{C}G_{0}+{}^{C}G_{1}+{}^{C}G_{2}+\cdots ~,\\ {}^{C}T(\tau )=&{}^{C}T_{0}+{}^{C}T_{1}+{}^{C}T_{2}+\cdots ~,\\ {}^{C}O(\tau )=&{}^{C}O_{0}+{}^{C}O_{1}+{}^{C}O_{2}+\cdots ~,\\ {}^{C}W(\tau )=&{}^{C}W_{0}+{}^{C}W_{1}+{}^{C}W_{2}+\cdots ~. \end{aligned} \end{aligned}$$

**NTDM**$_{CF}$: In view of Eq. (11) and initial conditions (3), we obtain

20 $$\begin{aligned} \begin{aligned} \frac{1}{f(\mu ,s,v)}\Big [N^{+}(V(\tau ))-\frac{40}{s}\Big ]=&N^{+}[\alpha -\epsilon \,V\,T-\vartheta \,V],\\ \frac{1}{f(\mu ,s,v)}\Big [N^{+}(G(\tau ))-\frac{10}{s}\Big ]=&N^{+}[\epsilon \,V\,T-\varepsilon _{1}\,G-\vartheta \,G],\\ \frac{1}{f(\mu ,s,v)}\Big [N^{+}(T(\tau ))-\frac{20}{s}\Big ]=&N^{+}[\varepsilon _{1}\,G+\varepsilon _{2}\,T\,O-(\vartheta +\rho )T],\\ \frac{1}{f(\mu ,s,v)}\Big [N^{+}(O(\tau ))-\frac{10}{s}\Big ]=&N^{+}[-\varepsilon _{2}\,T\,O-\vartheta \,O+\rho (1-\sigma )T],\\ \frac{1}{f(\mu ,s,v)}\Big [N^{+}(W(\tau ))-\frac{5}{s}\Big ]=&N^{+}[\sigma \,\rho \,T-\vartheta \,W]. \end{aligned} \end{aligned}$$

Operating the inverse NT on Eq. (20), we have

21 $$\begin{aligned} \begin{aligned} V(\tau )=&N^{-1}\Big [\frac{40}{s}+f(\mu ,s,v)N^{+}\Big [\alpha -\epsilon \,V\,T-\vartheta \,V\Big ]\Big ],\\ G(\tau )=&N^{-1}\Big [\frac{10}{s}+f(\mu ,s,v)N^{+}\Big [\epsilon \,V\,T-\varepsilon _{1}\,G-\vartheta \,G\Big ]\Big ],\\ T(\tau )=&N^{-1}\Big [\frac{20}{s}+f(\mu ,s,v)N^{+}\Big [\varepsilon _{1}\,G+\varepsilon _{2}\,T\,O-(\vartheta +\rho )T\Big ]\Big ],\\ O(\tau )=&N^{-1}\Big [\frac{10}{s}+f(\mu ,s,v)N^{+}\Big [-\varepsilon _{2}\,T\,O-\vartheta \,O+\rho (1-\sigma )T\Big ]\Big ],\\ W(\tau )=&N^{-1}\Big [\frac{5}{s}+f(\mu ,s,v)N^{+}\Big [\sigma \,\rho \,T-\vartheta \,W\Big ]\Big ]. \end{aligned} \end{aligned}$$

The nonlinear terms are included in Eq. (15) and also infinite series solution are included in Eq. (16).

By substituting Eqs. (15) and (16) into (21) to obtain

22 $$\begin{aligned} \begin{aligned} \sum _{k=0}^{\infty } V_{k}=&N^{-1}\Big [\frac{40}{s}\Big ]+N^{-1}\Big [f(\mu ,s,v)N^{+}\Big [\alpha -\epsilon \,\sum _{k=0}^{\infty } \sum _{j=0}^{k}V_{j}(T_{k-j})-\vartheta \,\sum _{k=0}^{\infty }V_{k}\Big ]\Big ],\\ \sum _{k=0}^{\infty } G_{k}=&N^{-1}\Big [\frac{10}{s}\Big ]+N^{-1}\Big [f(\mu ,s,v)N^{+}\Big [\epsilon \,\sum _{k=0}^{\infty } \sum _{j=0}^{k}V_{j}(T_{k-j})-\varepsilon _{1}\,\sum _{k=0}^{\infty }G_{k}-\vartheta \,\sum _{k=0}^{\infty }G_{k}\Big ]\Big ],\\ \sum _{k=0}^{\infty } T_{k}=&N^{-1}\Big [\frac{20}{s}\Big ]+N^{-1}\Big [f(\mu ,s,v)N^{+}\Big [\varepsilon _{1}\, \sum _{k=0}^{\infty }G_{k}+\varepsilon _{2}\,\sum _{k=0}^{\infty }\sum _{j=0}^{k}T_{j}(O_{k-j})-(\vartheta +\rho )\, \sum _{k=0}^{\infty }T_{k}\Big ]\Big ],\\ \sum _{k=0}^{\infty }O_{k}=&N^{-1}\Big [\frac{10}{s}\Big ]+N^{-1}\Big [f(\mu ,s,v)N^{+}\Big [\varepsilon _{2}\, \sum _{k=0}^{\infty }\sum _{j=0}^{k}T_{j}(O_{k-j})-\vartheta \,\sum _{k=0}^{\infty }O_{k}+\rho (1-\sigma )\, \sum _{k=0}^{\infty }T_{k}\Big ]\Big ],\\ \sum _{k=0}^{\infty }W_{k}=&N^{-1}\Big [\frac{5}{s}\Big ]+N^{-1}\Big [f(\mu ,s,v)N^{+} \Big [\sigma \,\rho \,\sum _{k=0}^{\infty }T_{k}-\vartheta \,\sum _{k=0}^{\infty }W_{k}\Big ]\Big ]. \end{aligned} \end{aligned}$$

From (22), we have

$$\begin{aligned} {}^{CF}V_{0}=40,~~ {}^{CF}G_{0}=10,~~ {}^{CF}T_{0}=20,~~ {}^{CF}O_{0}=10,~~ {}^{CF}W_{0}=5,~~ \end{aligned}$$

$$\begin{aligned} {}^{CF}V_{1}&= N^{-1}\Big [f(\mu ,s,v)N^{+}\Big [\alpha -\epsilon \,V_{0}\,T_{0}-\vartheta \,V_{0}\Big ]\Big ],\\ {}^{CF}G_{1}&= N^{-1}\Big [f(\mu ,s,v)N^{+}\Big [\epsilon \,V_{0}\,T_{0}-\varepsilon _{1}\,G_{0}-\vartheta \, G_{0}\Big ]\Big ],\\ {}^{CF}T_{1}&= N^{-1}\Big [f(\mu ,s,v)N^{+}\Big [\varepsilon _{1}\,G_{0}+\varepsilon _{2}\,T_{0}\,O_{0}-(\vartheta +\rho )\,T_{0}\Big ]\Big ],\\ {}^{CF}O_{1}&= N^{-1}\Big [f(\mu ,s,v)N^{+}\Big [-\varepsilon _{2}\,T_{0}\,O_{0}-\vartheta \,O_{0}+\rho (1-\sigma )\,T_{0}\Big ]\Big ],\\ {}^{CF}W_{1}&= N^{-1}\Big [f(\mu ,s,v)N^{+}\Big [\sigma \,\rho \,T_{0}-\vartheta \,W_{0}\Big ]\Big ],\\ {}^{CF}V_{2}&= N^{-1}\Big [f(\mu ,s,v)N^{+}\Big [\alpha -\epsilon \,(V_{0}\,T_{1}+V_{1}\,T_{0})-\vartheta \,V_{1}\Big ]\Big ],\\ {}^{CF}G_{2}&= N^{-1}\Big [f(\mu ,s,v)N^{+}\Big [\epsilon \,(V_{0}\,T_{1}+V_{1}\,T_{0}) -\varepsilon _{1}\,G_{1}-\vartheta \, G_{1}\Big ]\Big ],\\ {}^{CF}T_{2}&= N^{-1}\Big [f(\mu ,s,v)N^{+}\Big [\varepsilon _{1}\,G_{1}+\varepsilon _{2}\,(T_{0}\,O_{1}+T_{1}\,O_{0}) -(\vartheta +\rho )\,T_{1}\Big ]\Big ],\\ {}^{CF}O_{2}&= N^{-1}\Big [f(\mu ,s,v)N^{+}\Big [-\varepsilon _{2}\,(T_{0}\,O_{1}+T_{1}\,O_{0})-\vartheta \,O_{1}+\rho (1-\sigma )\,T_{1}\Big ]\Big ],\\ {}^{CF}W_{2}&= N^{-1}\Big [f(\mu ,s,v)N^{+}\Big [\sigma \,\rho \,T_{1}-\vartheta \,W_{1}\Big ]\Big ],\\&\vdots \end{aligned}$$

$$\begin{aligned} {}^{CF}V_{k+1}&= N^{-1}\Big [f(\mu ,s,v)N^{+}\Big [\alpha -\epsilon \, \sum _{j=0}^{k}V_{j}T_{k-j}-\vartheta \,V_{k}\Big ],~ k\ge 0, \\ {}^{CF}G_{k+1}&= N^{-1}\Big [f(\mu ,s,v)N^{+}\Big [\epsilon \,\sum _{j=0}^{k}V_{j}T_{k-j} -\varepsilon _{1}\,G_{k}-\vartheta \,G_{k}\Big ],~ k\ge 0, \\ {}^{CF}T_{k+1}&= N^{-1}\Big [f(\mu ,s,v)N^{+}\Big [\varepsilon _{1}\,G_{k}-\varepsilon _{2}\, \sum _{j=0}^{k}T_{j}O_{k-j}-(\vartheta +\rho )\,T_{k}\Big ],~ k\ge 0, \end{aligned}$$

23 $$\begin{aligned} \begin{aligned} {}^{CF}O_{k+1}&= N^{-1}\Big [f(\mu ,s,v)N^{+}\Big [-\varepsilon _{2}\,\sum _{j=0}^{k}T_{j}O_{k-j} -\vartheta \,O_{k}+\rho (1-\sigma )\,T_{k}\Big ],~ k\ge 0, \\ {}^{CF}W_{k+1}&= N^{-1}\Big [f(\mu ,s,v)N^{+}\Big [\sigma \, \rho \,T_{k}-\vartheta \,W_{k}\Big ],~ k\ge 0. \end{aligned} \end{aligned}$$

By substituting Eq. (23) into (16), we obtain the series solution as

24 $$\begin{aligned} \begin{aligned} {}^{CF}V(\tau )=&{}^{CF}V_{0}+{}^{CF}V_{1}+{}^{CF}V_{2}+\cdots ~,\\ {}^{CF}G(\tau )=&{}^{CF}G_{0}+{}^{CF}G_{1}+{}^{CF}G_{2}+\cdots ~,\\ {}^{CF}T(\tau )=&{}^{CF}T_{0}+{}^{CF}T_{1}+{}^{CF}T_{2}+\cdots ~,\\ {}^{CF}O(\tau )=&{}^{CF}O_{0}+{}^{CF}O_{1}+{}^{CF}O_{2}+\cdots ~,\\ {}^{CF}W(\tau )=&{}^{CF}W_{0}+{}^{CF}W_{1}+{}^{CF}W_{2}+\cdots ~. \end{aligned} \end{aligned}$$

**NTDM**$_{ABC}$: In view of Eq. (12) and initial conditions (3), we obtain

25 $$\begin{aligned} \begin{aligned} \frac{1}{h(\mu ,s,v)}\Big [N^{+}(V(\tau ))-\frac{40}{s}\Big ]=&N^{+}[\alpha -\epsilon \,V\,T-\vartheta \,V],\\ \frac{1}{h(\mu ,s,v)}\Big [N^{+}(G(\tau ))-\frac{10}{s}\Big ]=&N^{+}[\epsilon \,V\,T-\varepsilon _{1}\,G-\vartheta \,G],\\ \frac{1}{h(\mu ,s,v)}\Big [N^{+}(T(\tau ))-\frac{20}{s}\Big ]=&N^{+}[\varepsilon _{1}\,G+\varepsilon _{2}\,T\,O-(\vartheta +\rho )T],\\ \frac{1}{h(\mu ,s,v)}\Big [N^{+}(O(\tau ))-\frac{10}{s}\Big ]=&N^{+}[-\varepsilon _{2}\,T\,O-\vartheta \,O+\rho (1-\sigma )T],\\ \frac{1}{h(\mu ,s,v)}\Big [N^{+}(W(\tau ))-\frac{5}{s}\Big ]=&N^{+}[\sigma \,\rho \,T-\vartheta \,W]. \end{aligned} \end{aligned}$$

Operating the inverse NT on Eq. (25), we have

26 $$\begin{aligned} \begin{aligned} V(\tau )=&N^{-1}\Big [\frac{40}{s}+h(\mu ,s,v)N^{+}\Big [\alpha -\epsilon \,V\,T-\vartheta \,V\Big ]\Big ],\\ G(\tau )=&N^{-1}\Big [\frac{10}{s}+h(\mu ,s,v)N^{+}\Big [\epsilon \,V\,T-\varepsilon _{1}\,G-\vartheta \,G\Big ]\Big ],\\ T(\tau )=&N^{-1}\Big [\frac{20}{s}+h(\mu ,s,v)N^{+}\Big [\varepsilon _{1}\,G+\varepsilon _{2}\,T\,O-(\vartheta +\rho )T\Big ]\Big ],\\ O(\tau )=&N^{-1}\Big [\frac{10}{s}+h(\mu ,s,v)N^{+}\Big [-\varepsilon _{2}\,T\,O-\vartheta \,O+\rho (1-\sigma )T\Big ]\Big ],\\ W(\tau )=&N^{-1}\Big [\frac{5}{s}+h(\mu ,s,v)N^{+}\Big [\sigma \,\rho \,T-\vartheta \,W\Big ]\Big ]. \end{aligned} \end{aligned}$$

The nonlinear terms and also infinite series solution are included in NTDM$_C$.

By substituting Eqs. (15) and (16) into (26) to obtain

27 $$\begin{aligned} \begin{aligned} \sum _{k=0}^{\infty } V_{k}=&N^{-1}\Big [\frac{40}{s}\Big ]+N^{-1}\Big [h(\mu ,s,v)N^{+}\Big [\alpha -\epsilon \,\sum _{k=0}^{\infty }\sum _{j=0}^{k}V_{j}(T_{k-j})-\vartheta \,\sum _{k=0}^{\infty }V_{k}\Big ]\Big ],\\ \sum _{k=0}^{\infty } G_{k}=&N^{-1}\Big [\frac{10}{s}\Big ]+N^{-1}\Big [h(\mu ,s,v)N^{+}\Big [\epsilon \,\sum _{k=0}^{\infty }\sum _{j=0}^{k}V_{j}(T_{k-j})-\varepsilon _{1}\,\sum _{k=0}^{\infty }G_{k}-\vartheta \,\sum _{k=0}^{\infty }G_{k}\Big ]\Big ],\\ \sum _{k=0}^{\infty } T_{k}=&N^{-1}\Big [\frac{20}{s}\Big ]+N^{-1}\Big [h(\mu ,s,v)N^{+}\Big [\varepsilon _{1}\,\sum _{k=0}^{\infty }G_{k}+\varepsilon _{2}\,\sum _{k=0}^{\infty }\sum _{j=0}^{k}T_{j}(O_{k-j})-(\vartheta +\rho )\,\sum _{k=0}^{\infty }T_{k}\Big ]\Big ],\\ \sum _{k=0}^{\infty }O_{k}=&N^{-1}\Big [\frac{10}{s}\Big ]+N^{-1}\Big [h(\mu ,s,v)N^{+}\Big [\varepsilon _{2}\,\sum _{k=0}^{\infty }\sum _{j=0}^{k}T_{j}(O_{k-j})-\vartheta \,\sum _{k=0}^{\infty }O_{k}+\rho (1-\sigma )\,\sum _{k=0}^{\infty }T_{k}\Big ]\Big ],\\ \sum _{k=0}^{\infty }W_{k}=&N^{-1}\Big [\frac{5}{s}\Big ]+N^{-1}\Big [h(\mu ,s,v)N^{+}\Big [\sigma \,\rho \,\sum _{k=0}^{\infty }T_{k}-\vartheta \,\sum _{k=0}^{\infty }W_{k}\Big ]\Big ]. \end{aligned} \end{aligned}$$

From (27), we have

$$\begin{aligned} {}^{ABC}V_{0}=40,~~ {}^{ABC}G_{0}=10,~~ {}^{ABC}T_{0}=20,~~ {}^{ABC}O_{0}=10,~~ {}^{ABC}W_{0}=5,~~ \end{aligned}$$

$$\begin{aligned} {}^{ABC}V_{1}&= N^{-1}\Big [h(\mu ,s,v)N^{+}\Big [\alpha -\epsilon \,V_{0}\,T_{0}-\vartheta \,V_{0}\Big ]\Big ],\\ {}^{ABC}G_{1}&= N^{-1}\Big [h(\mu ,s,v)N^{+}\Big [\epsilon \,V_{0}\,T_{0}-\varepsilon _{1}\,G_{0}-\vartheta \, G_{0}\Big ]\Big ],\\ {}^{ABC}T_{1}&= N^{-1}\Big [h(\mu ,s,v)N^{+}\Big [\varepsilon _{1}\,G_{0}+\varepsilon _{2}\,T_{0}\,O_{0}-(\vartheta +\rho )\,T_{0}\Big ]\Big ],\\ {}^{ABC}O_{1}&= N^{-1}\Big [h(\mu ,s,v)N^{+}\Big [-\varepsilon _{2}\,T_{0}\,O_{0}-\vartheta \,O_{0}+\rho (1-\sigma )\,T_{0}\Big ]\Big ],\\ {}^{ABC}W_{1}&= N^{-1}\Big [h(\mu ,s,v)N^{+}\Big [\sigma \,\rho \,T_{0}-\vartheta \,W_{0}\Big ]\Big ],\\ {}^{ABC}V_{2}&= N^{-1}\Big [h(\mu ,s,v)N^{+}\Big [\alpha -\epsilon \,(V_{0}\,T_{1}+V_{1}\,T_{0})-\vartheta \,V_{1}\Big ]\Big ],\\ {}^{ABC}G_{2}&= N^{-1}\Big [h(\mu ,s,v)N^{+}\Big [\epsilon \,(V_{0}\,T_{1}+V_{1}\,T_{0}) -\varepsilon _{1}\,G_{1}-\vartheta \, G_{1}\Big ]\Big ],\\ {}^{ABC}T_{2}&= N^{-1}\Big [h(\mu ,s,v)N^{+}\Big [\varepsilon _{1}\,G_{1}+\varepsilon _{2}\,(T_{0}\,O_{1}+T_{1}\,O_{0}) -(\vartheta +\rho )\,T_{1}\Big ]\Big ],\\ {}^{ABC}O_{2}&= N^{-1}\Big [h(\mu ,s,v)N^{+}\Big [-\varepsilon _{2}\,(T_{0}\,O_{1}+T_{1}\,O_{0}) -\vartheta \,O_{1}+\rho (1-\sigma )\,T_{1}\Big ]\Big ],\\ {}^{ABC}W_{2}&= N^{-1}\Big [h(\mu ,s,v)N^{+}\Big [\sigma \,\rho \,T_{1}-\vartheta \,W_{1}\Big ]\Big ],\\&\vdots \end{aligned}$$

28 $$\begin{aligned} \begin{aligned} {}^{ABC}V_{k+1}&= N^{-1}\Big [h(\mu ,s,v)N^{+}\Big [\alpha -\epsilon \, \sum _{j=0}^{k}V_{j}T_{k-j}-\vartheta \,V_{k}\Big ],~ k\ge 0, \\ {}^{ABC}G_{k+1}&= N^{-1}\Big [h(\mu ,s,v)N^{+}\Big [\epsilon \,\sum _{j=0}^{k}V_{j}T_{k-j} -\varepsilon _{1}\,G_{k}-\vartheta \,G_{k}\Big ],~ k\ge 0, \\ {}^{ABC}T_{k+1}&= N^{-1}\Big [h(\mu ,s,v)N^{+}\Big [\varepsilon _{1}\,G_{k}-\varepsilon _{2}\, \sum _{j=0}^{k}T_{j}O_{k-j}-(\vartheta +\rho )\,T_{k}\Big ],~ k\ge 0, \\ {}^{ABC}O_{k+1}&= N^{-1}\Big [h(\mu ,s,v)N^{+}\Big [-\varepsilon _{2}\,\sum _{j=0}^{k}T_{j}O_{k-j} -\vartheta \,O_{k}+\rho (1-\sigma )\,T_{k}\Big ],~ k\ge 0, \\ {}^{ABC}W_{k+1}&= N^{-1}\Big [h(\mu ,s,v)N^{+}\Big [\sigma \,\rho \,T_{k}-\vartheta \,W_{k}\Big ],~ k\ge 0. \end{aligned} \end{aligned}$$

By substituting Eq. (28) into (16), we obtain the series solution as

29 $$\begin{aligned} \begin{aligned} {}^{ABC}V(\tau )=&{}^{ABC}V_{0} +{}^{ABC}V_{1}+{}^{ABC}V_{2}+\cdots ~,\\ {}^{ABC}G(\tau )=&{}^{ABC}G_{0}+{}^{ABC}G_{1}+{}^{ABC}G_{2}+\cdots ~,\\ {}^{ABC}T(\tau )=&{}^{ABC}T_{0}+{}^{ABC}T_{1}+{}^{ABC}T_{2}+\cdots ~,\\ {}^{ABC}O(\tau )=&{}^{ABC}O_{0}+{}^{ABC}O_{1}+{}^{ABC}O_{2}+\cdots ~,\\ {}^{ABC}W(\tau )=&{}^{ABC}W_{0}+{}^{ABC}W_{1}+{}^{ABC}W_{2}+\cdots ~. \end{aligned} \end{aligned}$$

## Existence and uniqueness

In this section we will present the existence and uniqueness results of the system (2), by considering the fractional derivative in the Caputo sense by making use of the approach given in^54^. Assume that

30 $$\begin{aligned} \begin{aligned} \Delta _{1}(\tau ,V,G,T,O,W)&=\alpha -\epsilon \,V\,T-\vartheta \,V, \\ \Delta _{2}(\tau ,V,G,T,O,W)&=\epsilon \,V\,T-\varepsilon _{1}\,G-\vartheta \,G, \\ \Delta _{3}(\tau ,V,G,T,O,W)&=\varepsilon _{1}\,G+\varepsilon _{2}\,T\,O-(\vartheta +\rho )T,\\ \Delta _{4}(\tau ,V,G,T,O,W)&=-\varepsilon _{2}\,T\,O-\vartheta \,O+\rho (1-\sigma )T,\\ \Delta _{5}(\tau ,V,G,T,O,W)&=\sigma \,\rho \,T-\vartheta \,W. \end{aligned} \end{aligned}$$

With the use of Eqs. (2) and (30), we can write

31 $$\begin{aligned} \begin{aligned} D^{\mu }_{\tau }V&=\Delta _{1}(\tau ,V,G,T,O,W),\\ D^{\mu }_{\tau }G&=\Delta _{2}(\tau ,V,G,T,O,W), \\ D^{\mu }_{\tau }T&=\Delta _{3}(\tau ,V,G,T,O,W),\\ D^{\mu }_{\tau }O&=\Delta _{4}(\tau ,V,G,T,O,W),\\ D^{\mu }_{\tau }W&=\Delta _{5}(\tau ,V,G,T,O,W). \end{aligned} \end{aligned}$$

Applying fractional integral and using initial conditions, we have

32 $$\begin{aligned} \begin{aligned} V(\tau )&=K_{1}+\frac{1}{\Gamma (\mu )}\int _{0}^{\tau }(\tau -\zeta )^{\mu -1}\Delta _{1}(\tau ,V,G,T,O,W)d\zeta ,\\ G(\tau )&=K_{2}+\frac{1}{\Gamma (\mu )}\int _{0}^{\tau }(\tau -\zeta )^{\mu -1}\Delta _{2}(\tau ,V,G,T,O,W), \\ T(\tau )&=K_{3}+\frac{1}{\Gamma (\mu )}\int _{0}^{\tau }(\tau -\zeta )^{\mu -1}\Delta _{3}(\tau ,V,G,T,O,W),\\ O(\tau )&=K_{4}+\frac{1}{\Gamma (\mu )}\int _{0}^{\tau }(\tau -\zeta )^{\mu -1}\Delta _{4}(\tau ,V,G,T,O,W),\\ W(\tau )&=K_{5}+\frac{1}{\Gamma (\mu )}\int _{0}^{\tau }(\tau -\zeta )^{\mu -1}\Delta _{5}(\tau ,V,G,T,O,W). \end{aligned} \end{aligned}$$

Let

33 $$\begin{aligned} X(\tau )=\left\{ \begin{array}{ll} &{}V(\tau )\\ &{}G(\tau )\\ &{}T(\tau )\\ &{}O(\tau )\\ &{}W(\tau ) \end{array} \right. , K=\left\{ \begin{array}{ll} &{}K_{1}\\ &{}K_{2}\\ &{}K_{3}\\ &{}K_{4}\\ &{}K_{5} \end{array} \right. , H(\tau ,X(\tau ))=\left\{ \begin{array}{ll} &{}\Delta _{1}(\tau ,V,G,T,O,W)\\ &{}\Delta _{2}(\tau ,V,G,T,O,W)\\ &{}\Delta _{3}(\tau ,V,G,T,O,W)\\ &{}\Delta _{4}(\tau ,V,G,T,O,W)\\ &{}\Delta _{5}(\tau ,V,G,T,O,W) \end{array} \right. . \end{aligned}$$

Using Eq. (33) in Eq. (32), we get

34 $$\begin{aligned} X(\tau )=K+\frac{1}{\Gamma (\mu )}\int _{0}^{\tau }(\tau -\zeta )^{\mu -1}H(\zeta ,X(\zeta ))d\zeta . \end{aligned}$$

Consider a Banach space $\Omega =C[0,T]\times C[0,T]$, with a norm

35 $$\begin{aligned} ||(V,G,T,O,W)||=\max \limits _{\tau \in [0,T]}[|V+G+T+O+W|]. \end{aligned}$$

Let $d:\Omega \rightarrow \Omega$ be a mapping defined as

36 $$\begin{aligned} d[X(\tau )]=K+\frac{1}{\Gamma (\mu )}\int _{0}^{\tau }(\tau -\zeta )^{\mu -1}H(\zeta ,X(\zeta ))d\zeta. \end{aligned}$$

Further, we impose some conditions on a nonlinear function as follows:

Condition (i): There exist conditions $C_{H}>0 ~~ \& ~~ Q_{H}>0$ such that

37 $$\begin{aligned} |H(\tau , X(\tau ))|\le C_{H} |X(\tau )|+Q_{H}. \end{aligned}$$

(ii) There exist conditions $L_{H}>0$ such that for each $\tilde{X}(\tau ), \tilde{X_{1}}(\tau )\in \Omega$ such that

38 $$\begin{aligned} |H(\tau , {\tilde{X}}(\tau ))-H(\tau , \tilde{X_{1}}(\tau ))|\le L_{H} |{\tilde{X}}(\tau )-\tilde{X_{1}}(\tau )|. \end{aligned}$$

### Theorem 2

Suppose that condition (i) holds. Then the system (31) has at least one solution.

### Proof

To show that operator *d* is bounded,

let $$\psi =\{X\in \psi |\beta \ge ||X||\}$$, where

39 $$\begin{aligned} \beta \ge \max \limits _{\tau \in [0,T]} \frac{K+Q_{H}\frac{\tau ^{\mu }}{\Gamma (\mu +1)}}{1-C_{H}\frac{\tau ^{\mu }}{\Gamma (\mu +1)}} \end{aligned}$$

is a closed and convex subset of $\Omega$. Now,

$$\begin{aligned} ||d(X)||&=\max \limits _{\tau \in [0,T]}\Big |K+\frac{1}{\Gamma (\mu )}\int _{0}^{\tau } (\tau -\zeta )^{\mu -1}H(\zeta ,X(\zeta ))d\zeta \Big |\\&\le |K|+\frac{1}{\Gamma (\mu )}\int _{0}^{\tau }(\tau -\zeta )^{\mu -1}|H(\zeta ,X(\zeta ))|d\zeta \\&\le |K|+\frac{1}{\Gamma (\mu )}\int _{0}^{\tau }(\tau -\zeta )^{\mu -1}(C_{H}|X(\tau )|+Q_{H})d\zeta \\ \end{aligned}$$

40 $$\begin{aligned} \le |K|+(C_{H}||X(\tau )||+Q_{H})\frac{\tau ^{\mu }}{\Gamma (\mu +1)}\le \beta. \end{aligned}$$

It means that $X\in \psi \Rightarrow d(\psi )\subseteq \psi$, which shows that *d* is bounded. Next, to show that *d* is completely continuous, let $\tau _{1}<\tau _{2}\in [0,T]$ and take

$$\begin{aligned} ||d(X)(\tau _{2})-d(X)(\tau _{1})||=\Big |\frac{1}{\Gamma (\mu )}\int _{0}^{\tau _{2}}(\tau _{2}-\zeta )^{\mu -1}H(\zeta ,X(\zeta ))d\zeta -\frac{1}{\Gamma (\mu )}\int _{0}^{\tau _{1}}(\tau _{1}-\zeta )^{\mu -1}H(\zeta ,X(\zeta ))d\zeta \Big | \end{aligned}$$

41 $$\begin{aligned} \le (\tau _{2}^{\mu }-\tau _{1}^{\mu })\frac{(C_{H}||X||+Q_{H})}{\Gamma (\mu +1)}. \end{aligned}$$

This shows that $||d(X)(\tau _{2}-d(x)(\tau _{1}))||\rightarrow 0$ as $\tau _{2}\rightarrow \tau _{1}$.

Hence, operator *d* is completely continuous by the Arzela Ascoli theorem. Thus the given system (31) has at least one solution by Schauder’s fixed-point theorem.

Next, To show the system (31) has unique solution by using Banach-fixed point theorem. $\square$

### Theorem 3

Suppose that condition (ii) holds. Then the system (31) has unique solution.

### Proof

Let $${\tilde{X}},\tilde{X_{1}}\in \Omega$$. Now

$$\begin{aligned} ||d(\tilde{X})-d(\tilde{X_{1}})||=\max \limits _{\tau \in [0,T]}\Big |\frac{1}{\Gamma (\mu )} \int _{0}^{\tau }(\tau -\zeta )^{\mu -1}H(\zeta ,\tilde{X}(\zeta ))d\zeta -\frac{1}{\Gamma (\mu )} \int _{0}^{\tau }(\tau -\zeta )^{\mu -1}H(\zeta ,\tilde{X_{1}}(\zeta ))d\zeta \Big |\\ \le \frac{\tau ^{\mu }}{\Gamma (\mu +1)}L_{H}|\tilde{X}-\tilde{X_{1}}|. \end{aligned}$$

Hence,*d* is the contraction. Using the Banach contraction theorem the system (31) has unique solution. $\square$

In similar lines, we can prove the uniqueness and existence for NTDM$_{CF}$ and NTDM$_{ABC}$ solutions of the system (2).

### Theorem 4

The $NTDM_C$ solution is convergent.

### Theorem 5

$NTDM_{CF}$ solution is convergent.

### Theorem 6

$NTDM_{ABC}$ solution is convergent.

## Illustrative example

In this section, the approximate solutions of nonlinear time fractional smoking model by applying three fractional derivatives are presented.

**NTDM**$_{C}$: We get the following NTDM$_C$ solutions as,

$$\begin{aligned} {}^{C}V_{0}(\tau )&=40,~~ {}^{C}G_{0}(\tau )=10,~~ {}^{C}T_{0}(\tau )=20,~~ {}^{C}O_{0}(\tau )=10,~~ {}^{C}W_{0}(\tau )=5,\\ {}^{C}V_{1}(\tau )&=-\frac{\tau ^{\mu } \,{\left( -\alpha +40\,\vartheta +800\,\epsilon \right) }}{{\Gamma }\left( 1+\mu \right) },\\ {}^{C}G_{1}(\tau )&=-\frac{10\,\tau ^{\mu }\,(\vartheta -80\,\epsilon +\varepsilon _{1})}{\Gamma (1+\mu )},\\ {}^{C}T_{1}(\tau )&=\frac{10\,\tau ^{\mu } \,\big (-2\,\rho -2\,\vartheta +20\,\varepsilon _{2} +\varepsilon _{1}\big )}{{\Gamma }\left( 1+\mu \right) },\\ {}^{C}O_{1}(\tau )&=-\frac{\tau ^{\mu } \,\big (200\,\varepsilon _{2} +10\,\vartheta +20\, \rho \,{\left( -1+\sigma \right) }\big )}{{\Gamma }\left( 1+\mu \right) },\\ {}^{C}W_{1}(\tau )&=-\frac{5\,\tau ^{\mu } \,\big (\vartheta -4\,\sigma \,\rho \big )}{{\Gamma }\left( 1+\mu \right) }, \end{aligned}$$

$$\begin{aligned} {}^{C}V_{2}(\tau )&=\frac{\alpha \, \tau ^{\mu }}{\Gamma (1+\mu )}-\frac{20\,\tau ^{2\mu }}{\Gamma (1+2\mu )} \Big (\epsilon \,(\alpha -40\,\rho -80\,\vartheta -800\,\epsilon +400\,\varepsilon _{2}+20\,\varepsilon _{1})\\&\quad +\vartheta \,(-\alpha +40\,\vartheta +800\,\epsilon )\Big ),\\ {}^{C}G_{2}(\tau )&=\frac{10\, \tau ^{2\mu }}{\Gamma (1+2\mu )}(\vartheta ^2+2\,\epsilon \,\alpha -80\, \epsilon \,\rho -240\,\epsilon \,\vartheta -1600\,\epsilon ^2+800\,\varepsilon _{2}\,\epsilon +2\, \varepsilon _{1}\,\vartheta -40\,\varepsilon _{1}\,\epsilon +\varepsilon _{1}^2),\\ {}^{C}T_{2}(\tau )&=-\frac{10\, \tau ^{2\mu }}{\Gamma (1+2\mu )}(-2\,\rho ^2-4\,\vartheta \,\rho -2\, \vartheta ^2+60\,\varepsilon _{2}\,\vartheta +40\,\varepsilon _{2}\,\sigma \,\rho +200\,\varepsilon _{2}^2 +\varepsilon _{1}\,\rho +2\,\varepsilon _{1}\,\eta \\&\quad -80\,\varepsilon _{1}\,\epsilon -10\,\varepsilon _{1}\,\varepsilon _{2}+\varepsilon _{1}^2),\\ {}^{C}O_{2}(\tau )&=\frac{10\, \tau ^{2\mu }}{\Gamma (1+2\mu )},(-2\,\rho ^2-4\,\vartheta \,\rho +\vartheta ^2+2\,\sigma \,\rho ^2+4\,\sigma \,\vartheta \,\rho +60\,\varepsilon _{2}\,\vartheta +20\, \varepsilon _{2}\,\sigma \,\rho +200\,\varepsilon _{2}^2\\&\quad +\varepsilon _{1}\,\rho -\varepsilon _{1}\,\sigma \,\rho -10\,\varepsilon _{1}\,\varepsilon _{2}),\\ {}^{C}W_{2}(\tau )&=\frac{5\, \tau ^{2\mu }}{\Gamma (1+2\mu )}(\vartheta ^2-4\,\sigma \,\rho ^2-8\,\sigma \,\vartheta \,\rho +40\, \varepsilon _{2}\,\sigma \,\rho +2\,\varepsilon _{1}\,\sigma \,\rho ). \end{aligned}$$

By continuing in this manner, one arrives at these approximate solutions

$$\begin{aligned} {}^{C}V(\tau )&=40-\frac{\tau ^{\mu } \,{\left( -\alpha +40\,\vartheta +800\,\epsilon \right) }}{{\Gamma } \left( 1+\mu \right) }+\frac{\alpha \, \tau ^{\mu }}{\Gamma (1+\mu )}-\frac{20\,\tau ^{2\mu }}{\Gamma (1+2\mu )} \Big (\vartheta \,(-\alpha +40\,\vartheta +800\,\epsilon )\\&\quad +\epsilon \,(\alpha -40\,\rho -80\,\vartheta -800\,\epsilon +400\,\varepsilon _{2}+20\,\varepsilon _{1})\Big ),\\ {}^{C}G(\tau )&=10-\frac{10\,\tau ^{\mu }\,(\vartheta -80\,\epsilon +\varepsilon _{1})}{\Gamma (1+\mu )}+\frac{10\, \tau ^{2\mu }}{\Gamma (1+2\mu )}(\vartheta ^2+2\,\epsilon \,\alpha -80\,\epsilon \,\rho -240\,\epsilon \,\vartheta -1600\,\epsilon ^2\\&\quad +800\,\varepsilon _{2}\,\epsilon +2\,\varepsilon _{1}\,\vartheta -40\,\varepsilon _{1}\,\epsilon +\varepsilon _{1}^2),\\ {}^{C}T(\tau )&=20+\frac{10\,\tau ^{\mu } \,\big (-2\,\rho -2\,\vartheta +20\,\varepsilon _{2} +\varepsilon _{1}\big )}{{\Gamma }\left( 1+\mu \right) }-\frac{10\, \tau ^{2\mu }}{\Gamma (1+2\mu )} (-2\,\rho ^2-4\,\vartheta \,\rho -2\,\vartheta ^2+60\,\varepsilon _{2}\,\vartheta \\&\quad +40\,\varepsilon _{2}\,\sigma \,\rho +200\,\varepsilon _{2}^2+\varepsilon _{1}\,\rho +2\, \varepsilon _{1}\,\vartheta -80\,\varepsilon _{1}\,\epsilon -10\,\varepsilon _{1}\,\varepsilon _{2}+\varepsilon _{1}^2),\\ {}^{C}O(\tau )&=10-\frac{\tau ^{\mu } \,\big (200\,\varepsilon _{2} +10\,\vartheta +20\, \rho \,{\left( -1+\sigma \right) }\big )}{{\Gamma }\left( 1+\mu \right) }+\frac{10\, \tau ^{2\mu }}{\Gamma (1+2\mu )}(-2\,\rho ^2-4\,\vartheta \,\rho +\vartheta ^2+2\,\sigma \,\rho ^2\\&\quad +4\,\sigma \,\vartheta \,\rho +60\,\varepsilon _{2}\,\vartheta +20\,\varepsilon _{2}\,\sigma \,\rho +200\, \varepsilon _{2}^2+\varepsilon _{1}\,\rho -\varepsilon _{1}\,\sigma \,\rho -10\,\varepsilon _{1}\,\varepsilon _{2}),\\ {}^{C}W(\tau )&=5-\frac{5\,\tau ^{\mu } \,\big (\vartheta -4\,\sigma \,\rho \big )}{{\Gamma }\left( 1+\mu \right) } +\frac{5\, \tau ^{2\mu }}{\Gamma (1+2\mu )}(\vartheta ^2-4\,\sigma \,\rho ^2-8\,\sigma \,\vartheta \,\rho +40\, \alpha _{2}\,\sigma \,\rho +2\,\varepsilon _{1}\,\sigma \,\rho ). \end{aligned}$$

**NTDM**$_{CF}$: We get the following NTDM$_{CF}$ solutions as,

$$\begin{aligned} {}^{CF}V_{0}(\tau )&=40,~~ {}^{CF}G_{0}(\tau )=10,~~ {}^{CF}T_{0}(\tau )=20,~~ {}^{CF}O_{0}(\tau )=10,~~ {}^{CF}W_{0}(\tau )=5,\\ {}^{CF}V_{1}(\tau )&=-{\left( 1-\mu +\mu \,\tau \right) }\,{\left( -\alpha +40\,\vartheta +800\,\epsilon \right) },\\ {}^{CF}G_{1}(\tau )&=-10\,{\left( 1-\mu +\mu \,\tau \right) }\,{\left( \vartheta -80\,\epsilon +\varepsilon _{1} \right) },\\ {}^{CF}T_{1}(\tau )&=10\,{\left( 1-\mu +\mu \,\tau \right) }\,{\left( -2\,\rho -2\,\vartheta +20\,\varepsilon _{2} +\varepsilon _{1} \right) },\\ {}^{CF}O_{1}(\tau )&=-10\,{\left( 1-\mu +\mu \,\tau \right) }\,{\left( -2\,\rho +\vartheta +2\,\sigma \,\rho +20\,\varepsilon _{2} \right) },\\ {}^{CF}W_{1}(\tau )&=-5\,{\left( 1-\mu +\mu \,\tau \right) }\,{\left( \vartheta -4\,\sigma \,\rho \right) },\\ {}^{CF}V_{2}(\tau )&=\alpha (1-\mu +\mu \,\tau )-20\,\Big (2(1-\mu )^2+4\,\mu \,\tau +\mu ^2\,\tau ^2\Big )\Big (\vartheta \,(-\alpha +40\,\vartheta +800\,\epsilon )\\&\quad +\epsilon \,(\alpha -40\,\rho -80\,\vartheta -800\,\epsilon +400\,\varepsilon _{2}+20\,\varepsilon _{1})\Big ),\\ {}^{CF}G_{2}(\tau )&=5\Big (2(1-\mu )^2+4\,\mu \,\tau +\mu ^2\,\tau ^2\Big )(\vartheta ^2+2\,\epsilon \,\alpha -80\,\epsilon \,\rho -240\,\epsilon \,\vartheta -1600\,\epsilon ^2\\&\quad +800\,\varepsilon _{2}\,\epsilon +2\,\varepsilon _{1}\,\vartheta -40\,\varepsilon _{1}\,\epsilon +\varepsilon _{1}^2),\\ {}^{CF}T_{2}(\tau )&=-5\Big (2(1-\mu )^2+4\,\mu \,\tau +\mu ^2\,\tau ^2\Big )(-2\,\rho ^2-4\,\vartheta \,\rho -2\,\vartheta ^2+60\,\varepsilon _{2}\,\vartheta +40\,\varepsilon _{2}\,\sigma \,\rho \\&\quad +200\,\varepsilon _{2}^2+\varepsilon _{1}\,\rho +2\,\varepsilon _{1}\,\vartheta -80\,\varepsilon _{1}\,\epsilon -10\,\varepsilon _{1}\,\varepsilon _{2}+\varepsilon _{1}^2),\\ {}^{CF}O_{2}(\tau )&=5\Big (2(1-\mu )^2+4\,\mu \,\tau +\mu ^2\,\tau ^2\Big )(-2\,\rho ^2-4\,\vartheta \,\rho +\vartheta ^2+2\,\sigma \,\rho ^2+4\,\sigma \,\rho \,\vartheta +60\,\varepsilon _{2}\,\vartheta \\&\quad +20\,\varepsilon _{2}\,\sigma \,\rho +200\,\varepsilon _{2}^2+\varepsilon _{1}\,\rho -\varepsilon _{1}\,\sigma \,\rho -10\,\varepsilon _{1}\,\varepsilon _{2}),\\ {}^{CF}W_{2}(\tau )&=5\Big ((1-\mu )^2+2\,\mu \,\tau \,(1-\mu )+\frac{\mu ^2\,\tau ^2}{\Gamma (3)}\Big )(\vartheta ^2-4\,\sigma \,\rho ^2-8\,\sigma \,\rho +40\,\varepsilon _{2}\,\sigma \,\rho +2\,\varepsilon _{1}\,\sigma \,\rho ). \end{aligned}$$

By continuing in the same manner, one arrives at these approximate solutions

$$\begin{aligned} {}^{CF}V(\tau )&=40-{\left( 1-\mu +\mu \,\tau \right) }\,{\left( -\alpha +40\,\vartheta +800\,\epsilon \right) }+\alpha (1-\mu +\mu \,\tau )-20\,\Big (2(1-\mu )^2+4\,\mu \,\tau +\mu ^2\,\tau ^2\Big )\\&\Big (\vartheta \,(-\alpha +40\,\vartheta +800\,\epsilon ) +\epsilon \,(\alpha -40\,\rho -80\,\vartheta -800\,\epsilon +400\,\varepsilon _{2}+20\,\varepsilon _{1})\Big ),\\ {}^{CF}G(\tau )&=10-10\,{\left( 1-\mu +\mu \,\tau \right) }\,{\left( \vartheta -80\,\epsilon +\varepsilon _{1} \right) }+5\Big (2(1-\mu )^2+4\,\mu \,\tau +\mu ^2\,\tau ^2\Big )\\&\quad (\vartheta ^2+2\,\epsilon \,\alpha -80\,\epsilon \,\rho -240\,\epsilon \,\vartheta -1600\,\epsilon ^2 +800\,\varepsilon _{2}\,\epsilon +2\,\varepsilon _{1}\,\vartheta -40\,\varepsilon _{1}\,\epsilon +\varepsilon _{1}^2),\\ {}^{CF}T(\tau )&=20+10\,{\left( 1-\mu +\mu \,\tau \right) }\,{\left( -2\,\rho -2\,\vartheta +20\,\varepsilon _{2} +\varepsilon _{1} \right) }-5\Big (2(1-\mu )^2+4\,\mu \,\tau +\mu ^2\,\tau ^2\Big )\\&\quad (-2\,\rho ^2-4\,\vartheta \,\rho -2\,\vartheta ^2+60\,\varepsilon _{2}\,\vartheta +40\,\varepsilon _{2}\,\sigma \,\rho +200\,\varepsilon _{2}^2+\varepsilon _{1}\,\rho +2\,\varepsilon _{1}\,\vartheta -80\,\varepsilon _{1}\,\epsilon -10\,\varepsilon _{1}\,\varepsilon _{2}+\varepsilon _{1}^2),\\ {}^{CF}O(\tau )&=10-10\,{\left( 1-\mu +\mu \,\tau \right) }\,{\left( -2\,\rho +\vartheta +2\,\sigma \,\rho +20\,\varepsilon _{2} \right) }+5\Big (2(1-\mu )^2+4\,\mu \,\tau +\mu ^2\,\tau ^2\Big )\\&\quad (-2\,\rho ^2-4\,\vartheta \,\rho +\vartheta ^2+2\,\sigma \,\rho ^2+4\,\sigma \,\rho \,\vartheta +60\,\varepsilon _{2}\,\vartheta +20\,\varepsilon _{2}\,\sigma \,\rho +200\,\varepsilon _{2}^2+\varepsilon _{1}\,\gamma -\varepsilon _{1}\,\sigma \,\rho -10\,\varepsilon _{1}\,\varepsilon _{2}),\\ {}^{CF}W(\tau )&=5-5\,{\left( 1-\mu +\mu \,\tau \right) }\,{\left( \vartheta -4\,\sigma \,\rho \right) }+5\Big ((1-\mu )^2+2\,\mu \,\tau \,(1-\mu )+\frac{\mu ^2\,\tau ^2}{\Gamma (3)}\Big )\\&\quad (\vartheta ^2-4\,\sigma \,\rho ^2-8\,\sigma \,\rho +40\,\varepsilon _{2}\,\sigma \,\rho +2\,\varepsilon _{1}\,\sigma \,\rho ). 
\end{aligned}$$

**NTDM**$_{ABC}$: We get the following NTDM$_{ABC}$ solutions as

$$\begin{aligned} {}^{ABC}V_{0}(\tau )&=40,~~ {}^{ABC}G_{0}(\tau )=10,~~ {}^{ABC}T_{0}(\tau )=20,~~ {}^{ABC}O_{0}(\tau )=10,~~ {}^{ABC}W_{0}(\tau )=5,\\ {}^{ABC}V_{1}(\tau )&=-\Big [1-\mu +\frac{\mu \,\tau ^{\mu }}{\Gamma (1+\mu )}\Big ]{\left( -\alpha +40\,\vartheta +800\,\epsilon \right) },\\ {}^{ABC}G_{1}(\tau )&=-10\,\Big [1-\mu +\frac{\mu \,\tau ^{\mu }}{\Gamma (1+\mu )}\Big ]{\left( \vartheta -80\,\epsilon +\varepsilon _{1} \right) },\\ {}^{ABC}T_{1}(\tau )&=10\,\Big [1-\mu +\frac{\mu \,\tau ^{\mu }}{\Gamma (1+\mu )}\Big ]{\left( -2\,\rho -2\,\vartheta +20\,\varepsilon _{2} +\varepsilon _{1} \right) },\\ {}^{ABC}O_{1}(\tau )&=-10\,\Big [1-\mu +\frac{\mu \,\tau ^{\mu }}{\Gamma (1+\mu )}\Big ]{\left( -2\,\rho +\vartheta +2\,\sigma \,\rho +20\,\varepsilon _{2} \right) },\\ {}^{ABC}W_{1}(\tau )&=-5\,\Big [1-\mu +\frac{\mu \,\tau ^{\mu }}{\Gamma (1+\mu )}\Big ](\vartheta -4\,\sigma \,\rho ),\\ {}^{ABC}V_{2}(\tau )&=\alpha \Big (1-\mu +\frac{\mu \,\tau ^{\mu }}{\Gamma (1+\mu )}\Big )-20\, \Big ((1-\mu )^2+2\,\mu \,(1-\mu )\frac{\tau ^{\mu }}{\Gamma (1+\mu )}+\frac{\mu ^{2}\,\tau ^{2\mu }}{\Gamma (1+2\mu )}\Big )\\&\Big (\epsilon (\alpha -40\,\rho -80\,\vartheta -800\,\epsilon +400\,\varepsilon _{2}+20\,\varepsilon _{1})+\vartheta (-\alpha -40\,\rho -80\,\vartheta +800\,\epsilon )\Big ),\\ {}^{ABC}G_{2}(\tau )&=10\Big [(1-\mu )^2+2\,\mu \,(1-\mu )\frac{\tau ^{\mu }}{\Gamma (1+\mu )}+\frac{\mu ^{2}\,\tau ^{2\mu }}{\Gamma (1+2\mu )}\Big ](\vartheta ^2+2\,\epsilon \,\alpha -80\,\epsilon \,\rho -240\,\epsilon \,\vartheta \\&\quad -1600\,\epsilon ^2+800\,\varepsilon _{2}\,\epsilon +2\,\varepsilon _{1}\,\vartheta -40\,\varepsilon _{1}\,\epsilon +\varepsilon _{1}^2),\\ {}^{ABC}T_{2}(\tau )&=-10\Big [(1-\mu )^2+2\,\mu \,(1-\mu )\frac{\tau ^{\mu }}{\Gamma (1+\mu )}+\frac{\mu ^{2}\,\tau ^{2\mu }}{\Gamma (1+2\mu )}\Big ](-2\,\rho ^2-4\,\vartheta \,\rho -2\,\vartheta ^2+60\,\varepsilon _{2}\,\vartheta \\&\quad +40\,\varepsilon _{2}\,\sigma \,\rho +200\,\varepsilon _{2}^2+\varepsilon _{1}\,\rho +2\,\varepsilon _{1}\,\vartheta -80\,\varepsilon _{1}\,\epsilon -10\,\varepsilon _{1}\,\varepsilon _{2}+\varepsilon _{1}^2),\\ {}^{ABC}O_{2}(\tau )&=10\Big [(1-\mu )^2+2\,\mu \,(1-\mu )\frac{\tau ^{\mu }}{\Gamma (1+\mu )}+\frac{\mu ^{2}\,\tau ^{2\mu }}{\Gamma (1+2\mu )}\Big ](-2\,\rho ^2-4\,\vartheta \,\rho +\vartheta ^2+2\,\sigma \,\rho ^2+4\,\sigma \,\vartheta \,\rho \\&\quad +60\,\varepsilon _{2}\,\vartheta +20\,\varepsilon _{2}\,\sigma \,\rho +200\,\varepsilon _{2}^2+\varepsilon _{1}\,\rho -\varepsilon _{1}\,\sigma \,\rho -10\,\varepsilon _{1}\,\varepsilon _{2}),\\ {}^{ABC}W_{2}(\tau )&=5\Big [(1-\mu )^2+2\,\mu \,(1-\mu )\frac{\tau ^{\mu }}{\Gamma (1+\mu )}+\frac{\mu ^{2}\,\tau ^{2\mu }}{\Gamma (1+2\mu )}\Big ](\vartheta ^2-4\,\sigma \,\rho ^2-8\,\sigma \,\vartheta \,\rho +40\,\alpha _{2}\,\sigma \,\rho +2\,\varepsilon _{1}\,\sigma \,\rho ). \end{aligned}$$

In the same manner, one arrives at these approximate solutions

$$\begin{aligned} {}^{ABC}V(\tau )&=40-\Big [1-\mu +\frac{\mu \,\tau ^{\mu }}{\Gamma (1+\mu )}\Big ]{\left( -\alpha +40\,\vartheta +800\,\epsilon \right) }+\alpha \Big (1-\mu +\frac{\mu \,\tau ^{\mu }}{\Gamma (1+\mu )}\Big )\\&\quad -20\, \Big ((1-\mu )^2+2\,\mu \,(1-\mu )\frac{\tau ^{\mu }}{\Gamma (1+\mu )}+\frac{\mu ^{2}\,\tau ^{2\mu }}{\Gamma (1+2\mu )}\Big )\\&\Big (\beta (\alpha -40\,\rho -80\,\vartheta -800\,\epsilon +400\,\varepsilon _{2}+20\,\varepsilon _{1})+\vartheta (-\alpha -40\,\rho -80\,\vartheta +800\,\epsilon )\Big ),\\ {}^{ABC}G(\tau )&=10-10\,\Big [1-\mu +\frac{\mu \,\tau ^{\mu }}{\Gamma (1+\mu )}\Big ]{\left( \vartheta -80\,\epsilon +\varepsilon _{1} \right) }+10\Big [(1-\mu )^2+2\,\mu \,(1-\mu )\frac{\tau ^{\mu }}{\Gamma (1+\mu )}+\frac{\mu ^{2}\,\tau ^{2\mu }}{\Gamma (1+2\mu )}\Big ]\\&\quad (\vartheta ^2+2\,\epsilon \,\alpha -80\,\epsilon \,\rho -240\,\epsilon \,\vartheta -1600\,\epsilon ^2+800\,\varepsilon _{2}\,\epsilon +2\,\varepsilon _{1}\,\vartheta -40\,\vartheta _{1}\,\epsilon +\varepsilon _{1}^2),\\ {}^{ABC}T(\tau )&=20+10\,\Big [1-\mu +\frac{\mu \,\tau ^{\mu }}{\Gamma (1+\mu )}\Big ]{\left( -2\,\rho -2\,\vartheta +20\,\varepsilon _{2} +\varepsilon _{1} \right) }\\&\quad -10\Big [(1-\mu )^2+2\,\mu \,(1-\mu )\frac{\tau ^{\mu }}{\Gamma (1+\mu )}+\frac{\mu ^{2}\,\tau ^{2\mu }}{\Gamma (1+2\mu )}\Big ](-2\,\rho ^2-4\,\vartheta \,\rho -2\,\vartheta ^2+60\,\varepsilon _{2}\,\vartheta \\&\quad +40\,\varepsilon _{2}\,\sigma \,\rho +200\,\varepsilon _{2}^2+\varepsilon _{1}\,\rho +2\,\varepsilon _{1}\,\vartheta -80\,\varepsilon _{1}\,\epsilon -10\,\varepsilon _{1}\,\varepsilon _{2}+\varepsilon _{1}^2),\\ {}^{ABC}O(\tau )&=10-10\,\Big [1-\mu +\frac{\mu \,\tau ^{\mu }}{\Gamma (1+\mu )}\Big ]{\left( -2\,\rho +\vartheta +2\,\sigma \,\rho +20\,\varepsilon _{2} \right) }\\&\quad +10\Big [(1-\mu )^2+2\,\mu \,(1-\mu )\frac{\tau ^{\mu }}{\Gamma (1+\mu )}+\frac{\mu ^{2}\,\tau ^{2\mu }}{\Gamma (1+2\mu )}\Big ](-2\,\rho ^2-4\,\vartheta \,\rho +\vartheta ^2+2\,\sigma \,\rho ^2+4\,\sigma \,\vartheta \,\rho \\&\quad +60\,\varepsilon _{2}\,\vartheta +20\,\varepsilon _{2}\,\sigma \,\rho +200\,\varepsilon _{2}^2+\varepsilon _{1}\,\rho -\varepsilon _{1}\,\sigma \,\rho -10\,\varepsilon _{1}\,\varepsilon _{2}),\\ {}^{ABC}W(\tau )&=5-5\,\Big [1-\mu +\frac{\mu \,\tau ^{\mu }}{\Gamma (1+\mu )}\Big ](\eta -4\,\sigma \,\rho )+5\Big [(1-\mu )^2+2\,\mu \,(1-\mu )\frac{\tau ^{\mu }}{\Gamma (1+\mu )}+\frac{\mu ^{2}\,\tau ^{2\mu }}{\Gamma (1+2\mu )}\Big ]\\&\quad (\vartheta ^2-4\,\sigma \,\rho ^2-8\,\sigma \,\vartheta \,\rho +40\,\varepsilon _{2}\,\sigma \,\rho +2\,\varepsilon _{1}\,\sigma \,\rho ). \end{aligned}$$

## Numerical results and discussion

**Figure 1:**
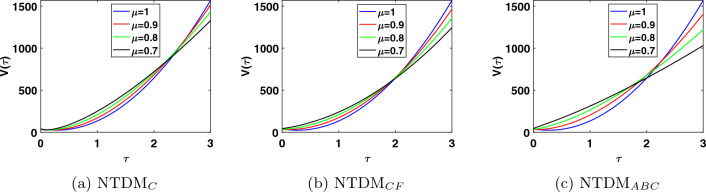
Approximate solution for potential somkers $V(\tau )$ with $\zeta =1$, different values $\mu$.

**Figure 2:**
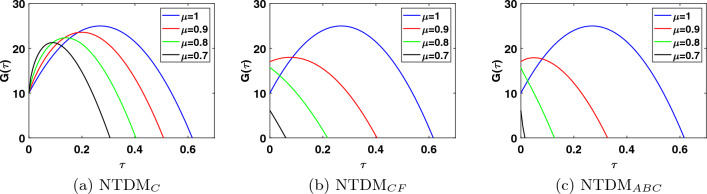
Approximate solution for occasional smokers $G(\tau )$ with $\zeta =1$, different values $\mu$.

**Figure 3:**
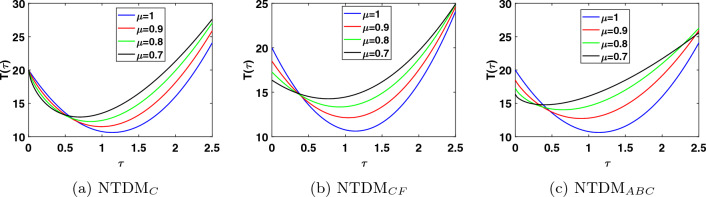
Approximate solution for smokers $T(\tau )$ with $\zeta =1$, different values $\mu$.

**Figure 4:**
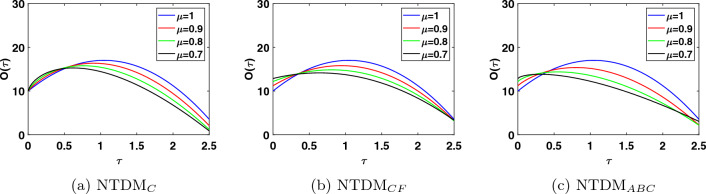
Approximate solution for temporarily quit smokers $O(\tau )$ with $\zeta =1$, different values $\mu$.

**Figure 5:**
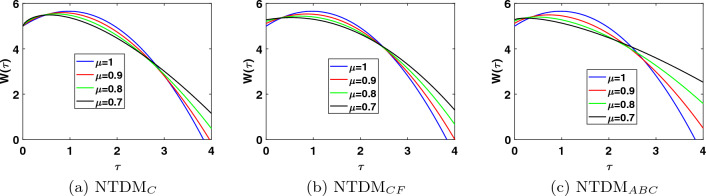
Approximate solution for permanently quit smokers $W(\tau )$ with $\zeta =1$, different values $\mu$.

This study presents the approximate solutions of a non-linear time fractional smoking epidemic model. The NTDM is utilised to investigate this model by considering the fractional derivative in Caputo, CF and ABC sense. The proposed method presents the outcomes of the smoking model by utilising tables and figures to observe the effects of the parameters. Graphical representation of the five compartments provides an analysis of the behaviour exhibited by each class, offering explanations for their respective actions. These simulations illustrate that a modification in value influenced the dynamics of the epidemic. This aids our understanding of the evolution of smoking patterns over time. The non-integer order has a minor impact on the dynamics of the epidemic, as demonstrated in Figs. 1, 2, 3, 4 and 5. Furthermore, this demonstrates that the methodology employed for fractional differential equations yields more accurate approximations for various fractional models. We utilised integer and fractional order for potential smokers (non-smokers), and it is evident that the number of non-smokers increases at fractional values of $\mu$. At the equilibrium point, one of the affected components (smokers) has a non-zero value, and its convergence can be observed for both integer and fractional values of $\mu$. Meanwhile, the other affected component converges to zero. Furthermore, it is evident that the infection rate diminishes as the fractional values of $\mu$ decrease. It is evident from Figs. 1 and 3 the total number of potential smokers $V(\tau )$ and smokers $T(\tau )$ increases over time and increases as the integer order $\mu$ goes down. From Figs. 2 and 4 it is clear that the number of occasional smokers $G(\tau )$ and temporarily quitters $O(\tau )$ increases over time and decreases as the integer order $\mu$ goes down. Figure 5 depicts the quantity of permanently quitters $W(\tau )$ increases over time and as the fractional parameter $\mu$ increases, but after a while the behavior reverses. Tables 2, 3, 4 and 5 present a comparison between the approximate solutions obtained using the present method and the existing methods in the literature, specifically LADM and q-HATM at various fractional order values $\mu$. Tables 6 and 7 displays a comparison of the proposed method solutions and the two established techniques in the literature for the classical derivative. The proposed method solution demonstrates a significant degree of agreement with these methods.

It is observed that the proposed method works well in producing approximations of solutions for the suggested mathematical model. We can infer from the results that the suggested approach is useful for comprehending behavior when using fractional derivatives.

**Table 2:** Approximate solution for potential smokers $V(\tau )$ at variation in $\mu$ and various values of $\tau$.

$\mu =0.8$						$\mu =0.9$				
$\tau$	NTDM$_{C}$	NTDM$_{CF}$	NTDM$_{ABC}$	q-HATM^55^	LADM^15^	NTDM$_{C}$	NTDM$_{CF}$	NTDM$_{ABC}$	q-HATM^55^	LADM^15^
0	40	34.1735	34.1735	40	40	40	32.9434	32.9434	40	40
0.1	28.2215	39.7982	46.1478	28.0338	28.6749	29.2565	31.9995	32.6841	29.1162	29.132
0.2	28.8861	48.0747	61.0339	28.5365	28.6749	26.28233	34.4118	37.5872	26.0052	26.0772
0.3	36.3236	59.0029	78.8113	35.8119	36.1378	28.8935	40.1803	46.6408	28.4733	28.6572
0.4	49.1258	72.5829	99.165	48.4484	49.0558	36.4457	49.3048	59.4646	35.8752	36.24
0.5	66.5397	88.8146	121.8705	65.6923	66.6838	48.56872	61.7855	75.8233	47.8402	48.4674

**Table 3:** Approximate solution for smokers $T(\tau )$ of variation in $\mu$ and various values of $\tau$.

$\mu =0.8$						$\mu =0.9$				
$\tau$	NTDM$_{C}$	NTDM$_{CF}$	NTDM$_{ABC}$	q-HATM^55^	LADM^15^	NTDM$_{C}$	NTDM$_{CF}$	NTDM$_{ABC}$	q-HATM^55^	LADM^15^
0	20	17.2836	17.2836	20	20	20	18.4969	18.4969	20	20
0.1	17.4502	16.4752	15.9919	17.4502	17.4386	17.9798	17.3332	17.0078	17.9798	17.9736
0.2	15.8889	15.7595	15.245	15.8891	15.8539	16.4515	16.2868	15.8974	16.4515	16.4298
0.3	14.7229	15.1366	14.7257	14.723	14.6555	15.1913	15.3578	14.9976	15.1913	15.1463
0.4	13.8383	14.6064	14.3716	13.8383	13.7315	14.1491	14.5462	14.2704	14.1491	14.0735
0.5	13.1806	14.1689	14.1523	13.1806	13.1863	13.2994	13.8519	13.6961	13.2994	13.1863

**Table 4:** Approximate solution for temporarily quit smokers $O(\tau )$ at various arbitrary orders of $\mu$ and $\tau$.

$\mu =0.8$						$\mu =0.9$				
$\tau$	NTDM$_{C}$	NTDM$_{CF}$	NTDM$_{ABC}$	q-HATM^55^	LADM^15^	NTDM$_{C}$	NTDM$_{CF}$	NTDM$_{ABC}$	q-HATM^55^	LADM^15^
0	10	12.1683	12.1683	10	10	11.2121	11.2121	10	10	10
0.1	12.0554	12.7899	13.1519	12.0554	11.9806	12.1359	12.3913	11.6331	11.276	11.592
0.2	13.2887	13.3297	13.6953	13.2887	13.0618	12.9562	13.2542	12.852	12.4241	12.7091
0.3	14.1876	13.7876	14.0492	14.1876	13.7537	13.6729	13.9372	13.8408	13.4443	13.5444
0.4	14.8466	14.1636	14.2642	14.8466	14.1591	14.2859	14.4716	14.6413	14.3365	14.1439
0.5	15.3112	14.4578	14.3649	15.3112	14.3278	14.7953	14.8736	15.2749	15.1008	14.5317

**Table 5:** Approximate solution for permanently quit smokers $W(\tau )$ at various fractional orders and $\tau$.

$\mu =0.8$						$\mu =0.9$				
$\tau$	NTDM$_{C}$	NTDM$_{CF}$	NTDM$_{ABC}$	q-HATM^55^	LADM^15^	NTDM$_{C}$	NTDM$_{CF}$	NTDM$_{ABC}$	q-HATM^55^	LADM^15^
0	5	5.2146	5.2146	5	5	5	5.1211	5.1211	5	5
0.1	5.2054	5.2738	5.3073	5.2054	7.4346	5.1636	5.2121	5.2369	5.1636	6.9295
0.2	5.3262	5.3241	5.3559	5.3262	8.9221	5.2841	5.2918	5.32	5.2841	8.3871
0.3	5.412	5.3656	5.385	5.412	10.0305	5.3803	5.3603	5.3842	5.3803	9.587
0.4	5.4726	5.3982	5.3997	5.4726	10.8685	5.4565	5.3175	5.4326	5.4565	10.5772
0.5	5.5127	5.4219	5.4024	5.5127	11.4884	5.5148	5.4636	5.4668	5.5148	11.3822

**Table 6:** Approximate solution of Smoking epidemic model with fixed $\mu =1$ at various values of $\tau$.

$\tau$	NTDM$_{C}$	NTDM$_{CF}$	NTDM$_{ABC}$	q-HATM^55^	LADM^15^	NTDM$_{C}$	NTDM$_{CF}$	NTDM$_{ABC}$	q-HATM^55^	LADM^15^
$V(\tau )$						$G(\tau )$				
0	40	40	40	40	40	10	10	10	10	10
0.1	30.8717	30.8717	30.8717	30.7667	30.7738	15.0559	15.0559	15.0559	15.0559	19.0721
0.2	25.8868	25.8868	25.8868	25.6668	25.7037	19.0756	19.0756	19.0756	19.0756	23.9787
0.3	25.0452	25.0452	25.0452	24.7002	24.8025	22.059	22.059	22.059	22.059	24.6999
0.4	28.347	28.347	28.347	27.867	28.0829	24.0063	24.0063	24.0063	24.0063	21.2122
0.5	35.7923	35.7923	35.7923	35.1672	35.5576	24.9173	24.9173	24.9173	24.9173	13.4978
$T(\tau )$						$O(\tau )$				
0	20	20	20	20	20	10	10	10	10	10
0.1	18.4244	18.4244	18.4244	18.4244	18.1142	11.276	11.276	11.276	11.276	11.2543
0.2	16.9938	16.9938	16.9938	16.9938	16.9805	12.4241	12.4241	12.4241	12.4241	12.3374
0.3	15.708	15.708	15.708	15.708	15.6782	13.4443	13.4443	13.4443	13.4443	13.2429
0.4	14.5671	14.5671	14.5671	14.5671	14.5141	14.3365	14.3365	14.3365	14.3365	13.9896
0.5	13.5711	13.5711	13.5711	13.5711	13.4883	15.1008	15.1008	15.1008	15.1008	14.5587

**Table 7:** Approximate outcomes of $W(\tau )$ for $\mu =1$ with various values of $\tau$.

$\tau$	NTDM$_{C}$	NTDM$_{CF}$	NTDM$_{ABC}$	q-HATM^55^	LADM^15^
0	5	5	5	5	5
0.1	5.1281	5.1281	5.1281	5.1281	6.6449
0.2	5.2423	5.2423	5.2423	5.2423	8.4294
0.3	5.3426	5.3426	5.3426	5.3426	10.3537
0.4	5.4291	5.4291	5.4291	5.4291	12.4177
0.5	5.5018	5.5018	5.5018	5.5018	14.6215

## Conclusion

Smoking increases the vulnerability of individuals to various perilous illnesses, such as oral, cervical, breast, and pancreatic cancers. The current framework utilises the natural transform decomposition approach for finding the approximate analytical solutions for the smoking epidemic model. The fractional derivatives included in the model under consideration are the Caputo, Caputo–Fabrizio, and Atangana–Baleanu–Caputo derivatives. In the context of the fractional-order smoking model, we analyse the concepts of reproduction number, endemic equilibrium point, and free disease equilibrium. The proposed method results demonstrate good agreement to limit smoking’s negative effects over various time periods and to eliminate a leading cause of death worldwide.Upon comparing the results to those of the q-HATM and LADM, it is observed that the outcomes align with the proposed approach, where $\mu$=1. Tables and graphs illustrate the characteristics of approximate solutions. The findings of this study will facilitate additional examination and the mitigation of diverse smoking-induced epidemics. In conclusion, we affirm that the proposed methodology is highly methodical and can be employed to analyse nonlinear fractional mathematical models that represent biological phenomena. Fractional calculus enables the development of novel mathematical modelling paradigms. Nonlinear differential equation systems were utilised to model a wide range of scientific and engineering problems. The suggested method can be applied to solve various types of models that arise in epidemiology, including those related to Ebola, Zika virus, and Monkey Pox, as well as in the fields of science and engineering.

## Data Availability

No datasets were generated or analysed during the current study
